# Are there socio-economic inequalities in utilization of predictive biomarker tests and biological and precision therapies for cancer? A systematic review and meta-analysis

**DOI:** 10.1186/s12916-020-01753-0

**Published:** 2020-10-23

**Authors:** Ruth P. Norris, Rosie Dew, Linda Sharp, Alastair Greystoke, Stephen Rice, Kristina Johnell, Adam Todd

**Affiliations:** 1 School of Pharmacy, Newcastle University, King George VI Building, King’s Road, Newcastle-upon-Tyne, NE1 7RU UK; 2 Population Health Sciences Institute, Newcastle University Centre for Cancer, Newcastle-upon-Tyne, UK; 3 Newcastle University Centre for Cancer, Newcastle-upon-Tyne, UK; 4 Health Economics Group and Evidence Synthesis Team, Population Health Sciences Institute, Newcastle University, Newcastle-upon-Tyne, UK; 5 Department of Medical Epidemiology and Biostatistics, Karolinska Institutet, Stockholm, Sweden

**Keywords:** Precision medicine, Molecular targeted therapy, Immunotherapy, Biological therapy, Pharmacogenomic testing, Drug utilization, Socio-economic factors, Meta-analysis

## Abstract

**Background:**

Novel biological and precision therapies and their associated predictive biomarker tests offer opportunities for increased tumor response, reduced adverse effects, and improved survival. This systematic review determined if there are socio-economic inequalities in utilization of predictive biomarker tests and/or biological and precision cancer therapies.

**Methods:**

MEDLINE, Embase, Scopus, CINAHL, Web of Science, PubMed, and PsycINFO were searched for peer-reviewed studies, published in English between January 1998 and December 2019. Observational studies reporting utilization data for predictive biomarker tests and/or cancer biological and precision therapies by a measure of socio-economic status (SES) were eligible. Data was extracted from eligible studies. A modified ISPOR checklist for retrospective database studies was used to assess study quality. Meta-analyses were undertaken using a random-effects model, with sub-group analyses by cancer site and drug class. Unadjusted odds ratios (ORs) and 95% confidence intervals (CIs) were computed for each study. Pooled utilization ORs for low versus high socio-economic groups were calculated for test and therapy receipt.

**Results:**

Among 10,722 citations screened, 62 papers (58 studies; 8 test utilization studies, 37 therapy utilization studies, 3 studies on testing and therapy, 10 studies without denominator populations or which only reported mean socio-economic status) met the inclusion criteria. Studies reported on 7 cancers, 5 predictive biomarkers tests, and 11 biological and precision therapies. Thirty-eight studies (including 1,036,125 patients) were eligible for inclusion in meta-analyses. Low socio-economic status was associated with modestly lower predictive biomarker test utilization (OR 0.86, 95% CI 0.71–1.05; 10 studies) and significantly lower biological and precision therapy utilization (OR 0.83, 95% CI 0.75–0.91; 30 studies). Associations with therapy utilization were stronger in lung cancer (OR 0.71, 95% CI 0.51–1.00; 6 studies), than breast cancer (OR 0.93, 95% CI 0.78–1.10; 8 studies). The mean study quality score was 6.9/10.

**Conclusions:**

These novel results indicate that there are socio-economic inequalities in predictive biomarker tests and biological and precision therapy utilization. This requires further investigation to prevent differences in outcomes due to inequalities in treatment with biological and precision therapies.

## Background

Traditional cancer treatments (chemotherapy, surgery, and radiotherapy) are subject to inequalities in utilization by socio-economic status [1]. These socio-economic inequalities have persisted over time and exist across cancers, healthcare systems, and treatments [2, 3]. Individuals with a lower socio-economic status are less likely to receive conventional treatments, and this may contribute to poorer cancer outcomes in this group [4].

Increasingly, systemic treatments targeted at cancer biology (e.g., tyrosine kinase inhibitors and monoclonal antibodies) are being integrated into cancer clinical care. These agents are expensive (immunotherapy can cost, in US dollars, $100,000 per patient annually) and may only have efficacy in selected sub-populations [5]. Hence, stratifying patients by molecular pathology to predict the likelihood of tumor response and adjusting therapy accordingly is now routinely recommended (see, for example, [6]). This move towards biological and precision therapies is reflected in the cancer drug development pipeline; for example, in 2019, 450 new cancer drug candidates were immunotherapies [7].

Socio-economic inequalities in biological and precision therapy utilization remain largely unexplored. Some speculate that using molecular information to target cancer treatment potentially provides a solution to current treatment inequalities [8]. Others argue that novel cancer therapies, because of their cost, disproportionately favor those with more resources and, therefore, may widen inequalities further [9–11].

As novel cancer therapies and their associated predictive biomarker tests offer opportunities for increased tumor response, reduced adverse effects, and improved survival, it is important to understand whether there are inequalities in their receipt [12, 13]. This systematic review and meta-analysis integrated the existing research to investigate the relationship between socio-economic status and utilization of biological and precision cancer therapies and their associated predictive biomarker tests.

## Methods

The review was registered with the international database of prospectively registered systematic reviews, PROSPERO (CRD42019140016), and is reported according to the Preferred Reporting Items for Systematic Reviews and Meta-Analyses (PRISMA) [14] (Additional file 1: Supplementary methods 1).

### Search strategy and study selection

Searches were performed in seven databases (MEDLINE, Embase, Scopus, CINAHL, Web of Science, PubMed, and PsycINFO) for articles published between January 1998 and December 2019. This time period reflects the licensing and approval of trastuzumab in the USA—considered a crucial time marker in the precision therapy field. Therapies of interest included the following: targeted therapy (targeting either oncogene addiction or synthetic lethality with activity restricted to tumors with appropriate biomarker status), biologics (where no predictive biomarker is included in the license), and immune checkpoint inhibitors. Therapies targeting hormone receptors were excluded as these agents have been in use since the early 1970s [15]. Search terms covering socio-economics status, tests, and therapies were developed; a full search strategy is available in Additional file 1: Supplementary methods 2. Reference lists of eligible articles were also reviewed.

The inclusion criteria for full-text papers, published 1998 onwards and written in English, were determined as follows in terms of PICOS (Population, Intervention, Comparison, Outcome, and Setting). *Population*: solid tumor cancer diagnosis (any age or sex). *Intervention*: receipt of either a predictive biomarker test or biological and precision therapy (or both). Studies reporting biological and precision therapies administered with an adjuvant (e.g., chemotherapy) were eligible as long as it was clear how many patients received the biological or precision therapy. Only predictive biomarker tests of pharmacological response to targeted treatment were included. *Comparison*: it was not a requirement that a comparator was reported but where noted, and the following comparator details were extracted—a clinical alternative, no biological and precision therapy and/or predictive biomarker tests, or no treatment. *Outcome*: utilization data reported by a socio-economic status measure (e.g., percent of persons living below the poverty line, median household income). *Setting*: retrospective or prospective observational design (including randomized controlled trials analyzed as observational cohorts). Full inclusion criteria are listed in Additional file 1: Supplementary methods 3.

Screening of titles and abstracts was conducted by one author (RN) only. All articles selected for full-text review were independently checked by a second author (AT). Disagreements were discussed and, if necessary, resolved with a third author (LS). Agreement between reviewers was excellent (κ = 0.93) [16].

### Data extraction and quality assessment

Data was extracted by one author (RN) and checked by another (RD). Disagreements were resolved through discussion with the review team (AG, AT, LS, and RD). In instances of missing or inconsistent data, study authors were contacted. Where there was no response, data was documented as not reported, or the paper excluded. In the event of multiple publications reporting identical or heavily overlapping study populations (e.g., same registry, cancer, stage, age group, and time period), data was extracted from the earliest publication, and where there was more than one publication from the same year, extraction first prioritized the publication reporting an income-based socio-economic measure and, second, one reporting multiple socio-economic measures. If more than one multivariable analysis was conducted, information was extracted from the most comprehensive adjusted model.

Data was extracted on author(s); publication year; country; data source; number in study population; cancer diagnosis time frame, patient age(s), cancer stage, and registry coverage; socio-economic measure and unit; numbers receiving predictive biomarker test/biological and precision therapy, overall and by socio-economic group (numerator and, where available, denominator); comparator(s) (where appropriate); and measures of association for not receiving testing/treatment by socio-economic status (e.g., ORs, 95% CIs, and *p* values). All eligible studies were quality appraised using a modified version of the ISPOR checklist for retrospective database studies. Focus in particular was paid to data sources, statistical results of interest, and generalizability of conclusions drawn [17]. The tool had ten features each scored as 0, 0.5, or 1 (Additional file 1: Supplementary methods 4). Appraisal was conducted independently by two authors (RN and RD), with disagreements resolved through discussion with a third author (AT), and consensus (AT).

### Synthesis of evidence

Data was synthesized using a summary of findings table. Where not reported, percentages utilizing biological and precision therapies and/or predictive biomarker test by socio-economic sub-group were calculated from data reported in the paper or supplied by authors, and unadjusted OR for low compared to high socio-economic status were computed for test/therapy receipt. Studies were heterogeneous in terms of outcome analyses (test/therapy receipt or non-receipt), socio-economic comparisons made, whether ORs (crude or adjusted) were reported, and the variables that any adjusted ORs were controlled for. Unadjusted ORs were therefore computed to enable inclusion of as many studies as possible in a consistent way. “Low” socio-economic status was defined as the lowest socio-economic sub-group in each article and “high” socio-economic status as the top sub-group.

Meta-analyses were performed using random-effects, Mantel-Haenszel methods. These assessed the likelihood of (i) test receipt and (ii) treatment receipt by low socio-economic status. Eligibility criteria for studies to be included in the meta-analysis were as follows: unadjusted low and high socio-economic utilization data for one measure of socio-economic status reported and an independent sampling frame (no data overlap with another study/paper). In the primary analyses, results relating to an income measure (or, failing that, education, or otherwise, the reported measure) were included. This reflected the dominance of USA studies within the evidence base, where there are cost implications for drug access [18]. Where multiple papers included study populations from the same or related databases that overlapped in terms of period of diagnosis/treatment, the publication reporting the largest total number of patients was entered into the meta-analysis.

For predictive biomarker tests, results were grouped by cancer site (breast, colorectal, lung, and melanoma). Those for biological and precision therapies were grouped by drug class (targeted therapy, biologic, and immunotherapy), while separate pre-specified sub-group analyses were conducted for breast cancer, lung cancer, and all other cancers (sub-grouped by cancer type: colorectal, head and neck, hepatobiliary, melanoma, mixed, renal cell). A final post hoc sub-group analysis was performed for the Surveillance, Epidemiology, and End Results program (SEER) versus non-SEER registry studies. Testing for sub-group differences was computed where appropriate. Two post hoc sensitivity analyses (one involving substituting included studies with those excluded due to overlapping sampling frames and the other exploring USA versus non-USA healthcare settings) were conducted to determine the robustness of the results. The *I*^2^ statistic was calculated to estimate the degree of statistical heterogeneity [19], and funnel plots were produced to assess publication bias in analyses of ten plus studies [20]. Statistical analyses were conducted using RevMan 5.3.

## Results

### Search results

**Fig. 1:**
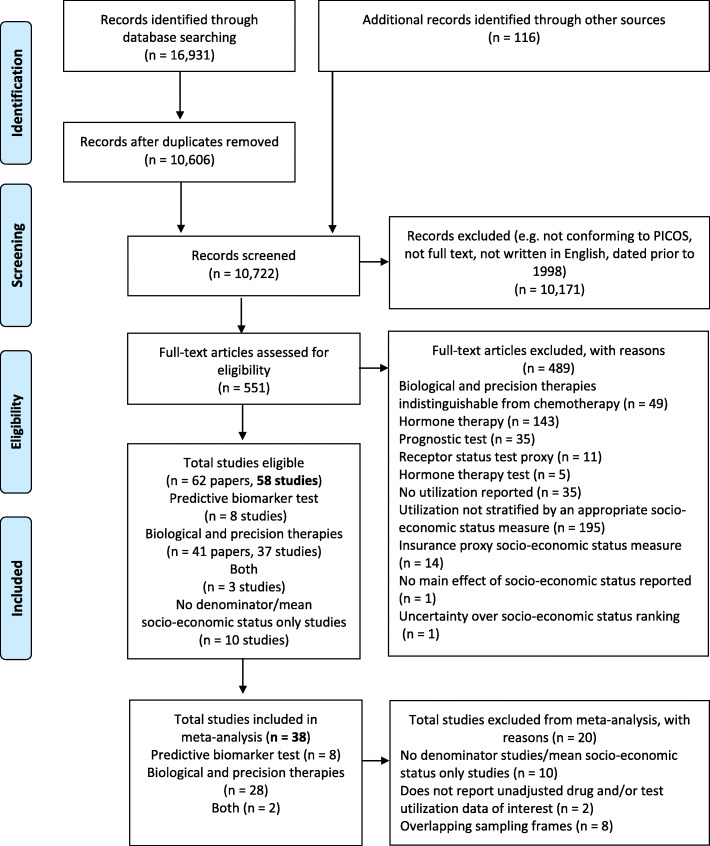
Study selection and exclusion according to the PRISMA statement

The search identified 17,047 citations. After removal of duplicates, titles and abstracts of 10,722 records were screened for eligibility. After title and abstract screening, 551 records progressed to full-text review. Overall, 62 papers (reporting 58 independent studies) met the inclusion criteria (Fig. 1) and were included in the review. Eight studies reported utilization data for predictive biomarker tests [21–28], thirty-seven studies (41 papers) reported utilization data for biological and precision therapies [29–65], and 3 studies reported both [66–68]. Ten papers (Additional file 1: Table S1) had no denominator populations or only reported an average measure of socio-economic status (e.g., mean household income), and were excluded from inclusion in the meta-analysis and are not discussed further [69–78].

### Study characteristics

The 48 included studies covered 7 cancers, 5 predictive biomarker tests, and 11 biological and precision therapy classifications, of which bevacizumab (12 studies) [41–45, 54–56, 58, 59, 64, 65] and trastuzumab (11 studies) [29–39] were most common. Most studies were in the USA (*n* = 42) [21, 22, 25–35, 40–68], and a majority analyzed SEER registry data (*n* = 27) [21, 22, 25, 29–33, 41, 42, 45, 47, 49, 50, 54–59, 61–64, 66–68] (Additional file 1: Fig. S1). Of the SEER data studies, 19 [29–32, 41, 42, 47, 49, 54–59, 61–64, 68] were SEER Medicare (i.e., included patients ≥ 65). The remaining studies were from Canada (4 studies) [23, 36–38], China (1 study) [39], and Ireland (1 study) [24]. Forty-six studies reported one or more area-based socio-economic status measure, and only two utilized individual-based measures [34, 68]. Six SES measures (poverty, income, education, employment, deprivation, and socio-economic status aggregate score) were reported. For nine studies, utilization was only available as percentages [24, 29, 32, 52, 54, 56, 61, 66, 67]. Study characteristics are summarized in Additional file 1: Table S2.

Seven papers, pertaining to four studies, reported the same data from the same registry [38, 43, 45, 79–82]. Sixteen papers (covering 8 studies) overlapped in their study populations (cancer site, stage, years of diagnosis time frames, patients’ age) [29–32, 36, 37, 41–44, 49, 50, 54, 55, 67, 68]. Two studies did not report unadjusted drug and/or test utilization data [40, 58]. This left 38 studies (including 1,036,125 patients) which were included in the meta-analysis [21–29, 31, 33–35, 37–39, 42, 44–49, 51–54, 56, 57, 59–66, 68].

### Quality appraisal

The 48 studies scored in the range 4–10, out of a possible 10 (mean = 6.9, median = 6.5) (Additional file 1: Table S3). Papers scored well regarding data source(s), study populations, and reporting socio-economic definition(s). Discussion of results with reference to the role of socio-economic status, statistical analysis with summary measures like OR, and explanations for confounder selection were often reported poorly.

### Predictive biomarker testing

**Fig. 2:**
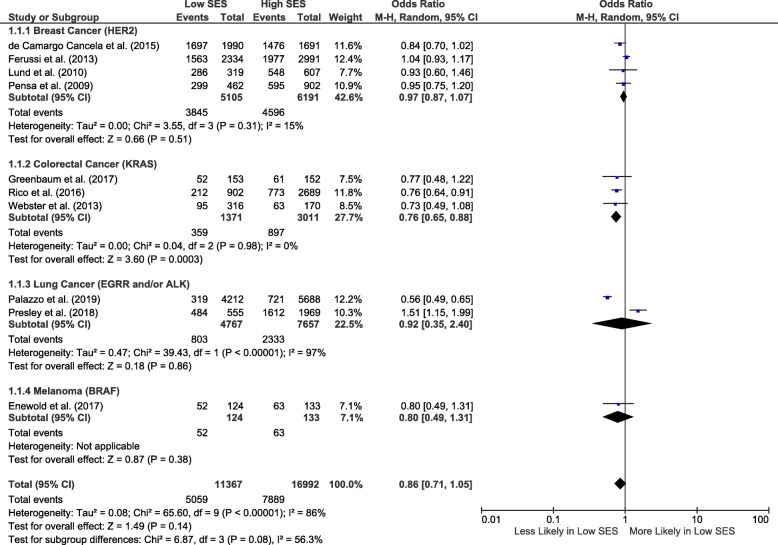
Forest plot showing predictive biomarker test utilization odds (sub-grouped by cancer type) for low compared to high socio-economic status. ALK, anaplastic lymphoma kinase; BRAF, proto-oncogene B-Raf; HER2, human epidermal growth factor receptor 2; KRAS, oncogene KRAS; SES, socio-economic status

Eleven studies reported data of interest for five predictive biomarker tests [21–28, 66–68]. Ten studies were included in the meta-analysis [21–28, 66, 68]. These covered the following cancers: breast (4 studies) [21–24], colorectal (3 studies) [25–27], melanoma (1 study) [66], and non-small cell lung (2 studies) [28, 68]. The pooled OR for predictive biomarker test receipt for low socio-economic status was 0.86 (95% CI 0.71–1.05; *I*^2^ = 86%; 10 studies) (Fig. 2). This pattern was consistent across cancer sub-groups (4 breast cancer studies, 2 lung cancer studies, and 1 melanoma study) but was only significant in colorectal cancer (0.76, 95% CI 0.65–0.88; 3 studies).

### Biological and precision therapies: primary analysis

**Fig. 3:**
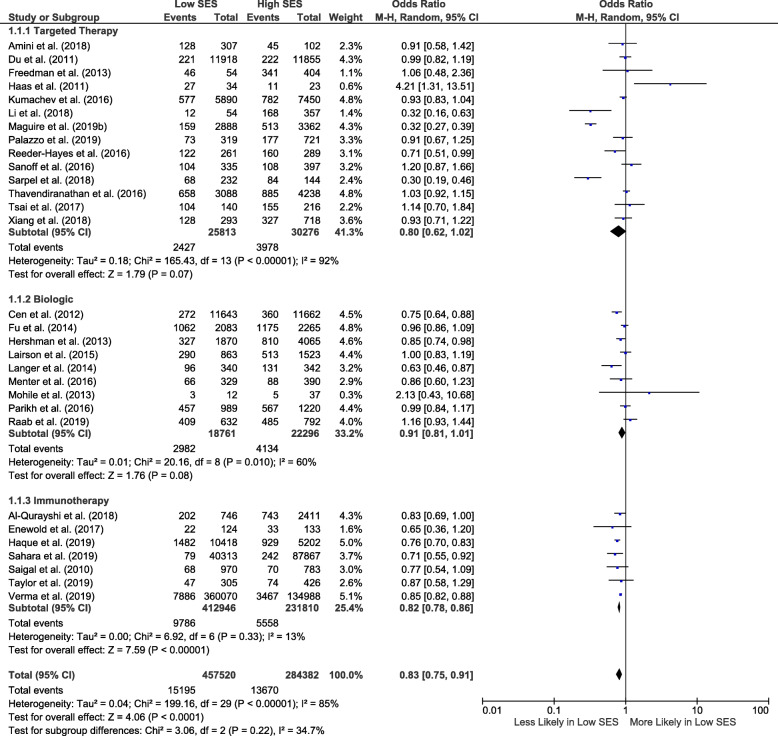
Forest plot showing biological and precision therapy utilization odds for all cancers (sub-grouped by drug class) for low compared to high socio-economic status. CI, confidence interval; SES, socio-economic status

Association of socio-economic status with biological and precision therapy receipt was reported in 40 studies [29–68]. Thirty of which were included in the meta-analysis [29, 31, 33–35, 37–39, 42, 44–49, 51–54, 56, 57, 59–66, 68]. The overall pooled OR for receipt of biological and precision therapy for patients from low socio-economic status was 0.83 (95% CI 0.75–0.91; *I*^2^ = 85%; 30 studies) (Fig. 3). Sub-group analysis suggested stronger associations with immunotherapy utilization (0.82, 95% CI 0.78–0.86; 7 studies) than other therapy classes (14 targeted therapy and 9 biological therapy studies), but the test for sub-group differences was not significant (Fig. 3). Sensitivity analyses which substituted included studies for excluded studies with overlapping sampling frames confirmed the robustness of results (0.80, 95% CI 0.72–0.88; *I*^2^ = 86%; 30 studies). Similar results were also observed in sensitivity analyses when only USA studies were considered (0.82, 95% CI 0.74–0.91, *I*^2^ = 85%, 27 studies). For full sensitivity analyses results, see Additional File 1: Fig. S2.

### Biological and precision therapies: sub-group analyses

**Fig. 4:**
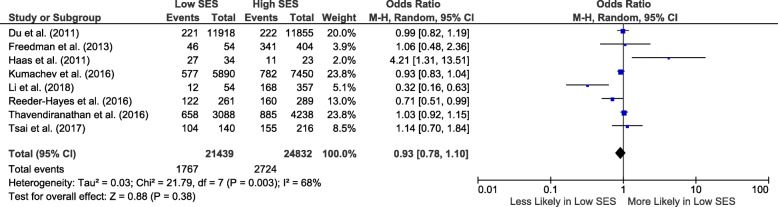
Forest plot showing biological and precision therapy utilization odds in breast cancer for low compared to high socio-economic status. CI, confidence interval; SES, socio-economic status

For breast cancer, 11 studies reported the association of socio-economic status with the human epidermal growth factor receptor 2 (HER2) targeting monoclonal antibody trastuzumab [29–39] and one with immunotherapy [40]. Eight studies were eligible for meta-analysis [29, 31, 33–35, 37–39]. The pooled OR for receipt of trastuzumab in those with low compared to high socio-economic status was 0.93 (95% CI 0.78–1.10; *I*^2^ = 68%) (Fig. 4).

**Fig. 5:**
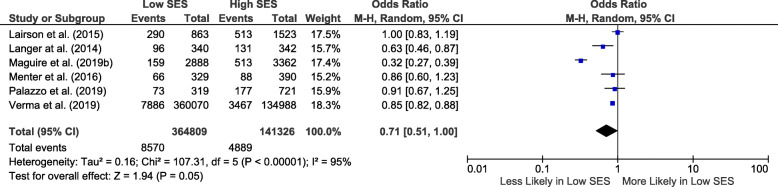
Forest plot showing biological and precision therapy utilization odds in lung cancer for low compared to high socio-economic status. CI, confidence interval; SES, socio-economic status

Nine lung cancer studies evaluated socio-economic status with biological and precision therapy receipt [41–47, 67, 68]. Four of these reported bevacizumab [41–44], 2 tyrosine kinase inhibitors [67, 68], 1 both bevacizumab and tyrosine kinase inhibitors [45], 1 immunotherapy [46], and 1 biological therapies (mostly bevacizumab) [47]. Six were eligible for meta-analysis [42, 44–47, 68], and the pooled OR for receipt of biological and precision therapies in those of low compared to high socio-economic status was 0.71 (95% CI 0.51–1.00; *I*^2^ = 95%) (Fig. 5).

Twenty studies reported data of interest for 6 other cancers: hepatobiliary (4 studies) [48–51], melanoma (3 studies) [52, 53, 66], colorectal (8 studies) [40, 54–60], renal cell carcinoma (1 study) [61], and head and neck cancer (2 studies) [62, 63]. A further two studies reported data on more than one cancer [64, 65]. Studies referenced the following 7 treatments: immunotherapy [40, 48, 52, 53, 60], bevacizumab [54–56, 58, 59, 64, 65], sorafenib [49–51], ipilimumab [66], targeted biologics [57], IL-2 [61], and cetuximab [62, 63]. Sixteen studies could be combined into meta-analyses [48, 49, 51–54, 56, 57, 59–66], giving a pooled OR for receipt of biological and precision therapies for low socio-economic status of 0.84 (95% CI 0.76–0.94; *I*^2^ = 73%) (Additional file 1: Fig. S3). The test for sub-group differences between breast, lung, and all other cancers was not significant (Additional file 1: Fig. S4).

## Discussion

This is the first systematic review and meta-analysis to examine whether there are inequalities in novel cancer therapeutics and/or associated testing use. Overall, the findings show that there are statistically significant socio-economic inequalities in biological and precision therapy utilization; those with a low socio-economic status were 17% less likely to be treated with precision therapies. An effect of similar magnitude was observed in test receipt, but did not achieve statistical significance.

The finding that differences are present in novel cancer treatments is consistent with previous systematic reviews documenting traditional treatment inequalities [2, 3]. Similar socio-economic inequalities have also been observed across the cancer care pathway (from screening [83], to diagnosis [84], and timeliness of referral and treatment receipt [85] through to survival [86]). Combined, this suggests that low socio-economic status remains a barrier to treatment access and cancer care, despite advances in treatment.

The strength of socio-economic inequalities varied with cancer type: the effect estimate for receipt of biological and precision therapies was stronger for lung cancer (incidence of which is related to low socio-economic status) than other cancers. It is not clear why this is so, although the risk of some cancers (including lung) is associated with health behaviors (e.g., smoking) [87]. It is possible that these health behaviors, alongside other factors (which, themselves may be a consequence of the health behaviors), such as multi-morbidity, could influence a healthcare professional’s decision to offer or initiate, or a patient’s choice to receive, cancer treatment [88]. While such individual behavioral factors warrant further investigation, they need contextualizing within the wider determinants of health (i.e., the social, economic, cultural, and clinical level factors) which are also associated with known treatment barriers [89].

The socio-economic inequalities in testing and therapy utilization in breast cancer were less pronounced, despite the majority of research focusing on this cancer. This finding, along with a previous systematic review concluding equivocal associations between socio-economic status and trastuzumab uptake [90], suggests that low socio-economic status may be less of a treatment barrier in breast cancer, at least as far as newer therapies are concerned. One possible explanation for this may be that breast cancer sub-type differentiation and the practice of hormone receptor status testing, and basing treatment on these results, are well established and routinely embedded in clinical practice (originating in the 1970s following the discovery of the estrogen receptor) [91]. Hence, our findings support the wider concept of the inverse equity hypothesis [92]: that is, that while new interventions may temporarily widen inequalities by disproportionately favoring those with resources enabling priority access, over time this narrows as treatment access “trickles down” and becomes standard clinical practice [93, 94].

In relation to predictive biomarker testing more generally, the observation that there is reduced utilization with respect to socio-economic status builds on previously documented relationships between factors associated with socio-economic status and test receipt (e.g., negative association between smoking and epidermal growth factor receptor (EGFR) and anaplastic lymphoma kinase (ALK) abnormalities) [95]. Previous work also highlights that test patterns vary temporally and spatially [96, 97], as well as with respect to patient demographics (e.g., age) [98]. This suggests that testing access is complex. Nevertheless, the observation that low socio-economic status may reduce access to testing has important implications. First, utilization barriers occur at points other than just therapy receipt, a finding echoed by Cancer Research UK who highlighted that many colorectal and non-small cell lung cancer patients potentially eligible for targeted treatments did not receive molecular testing [99]. Second, if multiple barriers to novel therapy utilization exist, then sophisticated solutions are likely required to prevent cancer inequalities widening further. In the first instance, further monitoring of inequalities is required. However, given the rapidly evolving nature of the precision oncology field, and the fact that routine datasets generally lack good data on newly licensed therapies and tests, an appreciation of how such information might be captured in future observational studies is required, especially those that are large-scale and population-based. Analysis of new data sources, rich in biological and precision therapies (e.g., UK’s Systemic Anti-cancer Therapy dataset) or predictive biomarker test information (e.g., USA’s Flatiron Health electronic healthcare records database), may provide the first steps here. Encouragement of data collection to enable audit of treatment access would inform development of solutions to respond to any inequalities noted (e.g., low-income assistance programs, investigating access barriers in problem areas) [9].

This is the first comprehensive meta-analysis on this important and growing area of practice, and brings together data on over 1 million patients. Despite this, the study does have several limitations. First, there are challenges comparing studies reporting different measures of socio-economic status. There was no one consistent measure used, and even when studies appeared to use the same measure (e.g., income), how the variable was categorized (e.g., what was considered “high”; number of sub-groups considered) differed. For most studies, there was considerable variation between what was classified as “high” and “low” socio-economic status, meaning that true differences were unlikely to be attenuated by a lack of variability. However, almost all studies used area-based socio-economic measures, so the ecological fallacy in inference is a risk. Secondly, determining OR from raw data disregards adjustments for confounders; this along with variations in study sampling frames may in part explain the high heterogeneity observed. It also means that the possibility cannot be entirely excluded that any associations seen in the meta-analyses could be explained by uncontrolled confounding. Third, single reviewer title and abstract screening, while considered acceptable by the Cochrane Collaboration [100], may have erroneously excluded relevant studies. Finally, any conclusions drawn here are time specific and may not fully reflect all inequalities present within the system.

The review also highlights limitations in the evidence base. For example, sub-group analyses require care in interpretation where study numbers are small. The majority of studies reported data from non-universal healthcare systems and recorded in SEER Medicare registries. As the relevance of socio-economic indicators varies across the life course, measures such as median household income may be less meaningful in retired SEER populations [101]. In such circumstances, eligibility for Medicare may be more important in addressing one of the most important barriers to care in the USA—that of having health insurance. Similarly, as employment is often tied to insurance coverage in non-universal healthcare systems like the USA, this choice of socio-economic indicator could be an additional factor related to utilization outcomes in the under 65 age group other than income alone. The generalizability of conclusions drawn to patients outside the USA and age groups younger than 65 years must be questioned. Having said this, studies from other countries documented similar patterns in inequality [24, 37, 39]. Moving forward, consideration of data from other registries (e.g., Scandinavian datasets known to be rich in socio-economic detail) would be valuable. The SEER registry also underrepresents minority populations. This limitation may be important given the links between ethnicity and genetics [102–104]. Despite these limitations, among all analyses, there was no clear observable evidence of publication bias (Additional file 1: Fig. S5).

Future research should focus on investigating the reasons for inequalities around these novel therapies. Consideration of testing as a treatment barrier requires prioritization, and work investigating clinician, patient, and family roles in decision-making around testing and treatment receipt is crucial. This is even more pertinent given the projected increases in panel sequencing testing costs and the growing number of therapeutic agents entering clinical practice. To aid further work in this area, it would be helpful if researchers critically evaluated the relationships between the different measures of socio-economic status in healthcare utilization research: for example, individual versus population measures, single versus aggregate measures, or the various single measures such as education or income. Doing so acknowledges that there is not one standardized, superior socio-economic measure to select. Rather, that as all indicators have limitations and the constraints of current dataset access may restrict the feasibility of further measurement, the magnitude of inequalities observed as well as the ability to make cross-study comparisons requires contextualizing in any future findings. From a practice perspective, policymakers and clinicians need to be aware of the potential barriers to biological and precision therapy beyond patients’ tumor molecular profiles. Revising guidelines to include a focus on reducing inequalities would assist with such prioritization.

## Conclusions

There are socio-economic inequalities in the utilization of both predictive biomarker tests as well as biological and precision cancer therapies. This requires further investigation to prevent differences in outcomes due to inequalities in treatment with biological and precision therapies.

## Supplementary information

**Additional file 1: Supplementary methods 1**, PRISMA checklist. **Supplementary methods 2**, search strategy. **Supplementary methods 3**, inclusion/exclusion decision trees. **Supplementary methods 4**, quality appraisal tool. **Table S1**, no denominator/mean socio-economic status only study characteristics. **Fig. S1**, SEER versus non-SEER registry studies forest plot and funnel plot. **Table S2**, included studies characteristics. **Table S3**, quality appraisal results breakdown. **Fig. S2**, sensitivity analyses. **Fig. S3**, all other cancers forest plot and funnel plot. **Fig. S4**, breast, lung and all other cancers forest plot and funnel plot. **Fig. S5**, additional funnel plots.

## Data Availability

All data generated or analyzed during this study are included in this published article and its supplementary information files.

## Abbreviations

ALK: Anaplastic lymphoma kinase
BRAF: Proto-oncogene B-Raf
CI/CIs: 95% Confidence interval
EGFR: Epidermal growth factor receptor
HER2: Human epidermal growth factor receptor 2
KRAS: Oncogene KRAS
OR/ORs: Odds ratio
PICOS: Population, Intervention, Comparison, Outcome, and Setting
PRISMA: Preferred Reporting Items for Systematic Reviews and Meta-analyses
SEER: Surveillance, Epidemiology, and End Results program
SES: Socio-economic status

## References

[1] Macmillan. Health inequalities: time to talk. 2019. https://macmillan.org.uk/assets/health-inequalities-paper-april-2019.pdf. Accessed 15 Mar 2020.

[2] Forrest LF, Adams J, Wareham H, Rubin G, White M (2013). Socioeconomic inequalities in lung cancer treatment: systematic review and meta-analysis. PLoS Med.

[3] Aarts MJ, Lemmens VEPP, Louwman MWJ, Kunst AE, Coebergh JWW (2010). Socioeconomic status and changing inequalities in colorectal cancer? A review of the associations with risk, treatment and outcome. Eur J Cancer.

[4] Halpern MT (2015). Cancer disparities research: it is time to come of age. Cancer..

[5] Dranitsaris G, Zhu X, Adunlin G, Vincent MD (2018). Cost effectiveness vs affordability in the age of immuno-oncology cancer drugs. Expert Rev Pharmacoecon Outcomes Res.

[6] NCCN. Clinical practice guidelines in oncology: non-small cell lung cancer. 2020. http://www.nccn.org/professionals/physician_gls/pdf/nscl.pdf. Accessed 23 Mar 2020.

[7] IQVIA Institute. Global oncology trends 2019 therapeutics, clinical development and health system implications institute report. 2019. https://www.iqvia.com/-/media/iqvia/pdfs/institute-reports/global-oncology-trends-2019.pdf?_=1587463035271. Accessed 14 Mar 2020.

[8] McClellan KA, Avard D, Simard J, Knoppers BM (2013). Personalized medicine and access to health care: potential for inequitable access?. Eur J Hum Genet.

[9] Ward MM (2012). Personalized therapeutics: a potential threat to health equity. J Gen Intern Med.

[10] James JE (2014). Personalised medicine, disease prevention, and the inverse care law: more harm than benefit?. Eur J Epidemiol.

[11] Huey RW, Hawk E, Offodile AC (2019). Mind the gap: precision oncology and its potential to widen disparities. J Onco Practice.

[12] Schwaederle M, Zhao M, Lee JJ, Eggermont AM, Schilsky RL, Mendelsohn J (2015). Impact of precision medicine in diverse cancers: a meta-analysis of phase II clinical trials. J Clin Oncol.

[13] Mok TS, Wu Y-L, Thongprasert S, Yang C-H, Chu D-T, Saijo N (2009). Gefitinib or carboplatin–paclitaxel in pulmonary adenocarcinoma. N Engl J Med.

[14] Moher D, Liberati A, Tetzlaff J, Altman DG, Group P (2009). Preferred reporting items for systematic reviews and meta-analyses: the PRISMA statement. PLoS Med.

[15] Ross JS. Targeted therapies for cancer. In: Gingsburg GS, Willard HF, editors. Essentials of genomic and personalized medicine. 1st ed. Amsterdam; Boston; London: Elsevier; 2010. p. 532–547.

[16] Higgins J, Deek JJ. Chapter 7: selecting studies and collecting data. In: Higgins JPT, Green S, editors. Cochrane handbook for systematic reviews of interventions. Version 510 [updated March 2011]. Chichester: Wiley; 2011.

[17] Motheral B, Brooks J, Clark MA, Crown WH, Davey P, Hutchins D (2003). A checklist for retrospective database studies—report of the ISPOR task force on retrospective databases. Value Health.

[18] Braveman PA, Cubbin C, Egerter S, Chideya S, Marchi KS, Metzler M (2005). Socioeconomic status in health research one size does not fit all. JAMA..

[19] Higgins JPT, Thompson SG (2002). Quantifying heterogeneity in a meta-analysis. Stat Med.

[20] Egger M, Smith GD, Schneider M, Minder C (1997). Bias in meta-analysis detected by a simple, graphical test. BMJ..

[21] Pensa M, Swede H, Brockmeyer JA, Gregorio DI (2009). Patterns of HER2 testing in the management of primary breast cancer. Cancer Epidemiol.

[22] Lund MJ, Butler EN, Hair BY, Ward KC, Andrews JH, Oprea-Ilies G (2010). Age/race differences in HER2 testing and in incidence rates for breast cancer triple subtypes. Cancer..

[23] Ferrusi IL, Earle CC, Trudeau M, Leighl NB, Pullenayegum E, Khong H (2013). Closing the personalized medicine information gap: HER2 test documentation practice. Am J Manag Care.

[24] de Camargo CM, Comber H, Sharp L (2015). Which women with breast cancer do, and do not, undergo receptor status testing? A population-based study. Cancer Epidemiol.

[25] Greenbaum A, Wiggins C, Meisner AL, Rojo M, Kinney AY, Rajput A. KRAS biomarker testing disparities in colorectal cancer patients in New Mexico. Heliyon. 2017;(3, 11):e00448-e. 10.1016/j.heliyon.2017.e00448 PMC5701808 29202108

[26] Rico A, Pollack LA, Thompson TD, Hsieh M-C, Wu X-C, Karlitz JJ (2016). KRAS testing and first-line treatment among patients diagnosed with metastatic colorectal cancer using population data from ten national program of cancer registries in the United States. J Cancer Res Ther (Manch)..

[27] Webster J, Kauffman TL, Feigelson HS, Pawloski PA, Onitilo AA, Potosky AL (2013). KRAS testing and epidermal growth factor receptor inhibitor treatment for colorectal cancer in community settings. Cancer Epidemiol Biomark Prev.

[28] Presley CJ, Tang D, Soulos PR, Chiang AC, Longtine JA, Adelson KB (2018). Association of broad-based genomic sequencing with survival among patients with advanced non–small cell lung cancer in the community oncology setting. JAMA..

[29] Du XL, Xia R, Burau K, Liu C-C (2011). Cardiac risk associated with the receipt of anthracycline and trastuzumab in a large nationwide cohort of older women with breast cancer, 1998–2005. Med Oncol.

[30] Vaz-Luis I, Lin NU, Keating NL, Barry WT, Lii H, Winer EP (2015). Racial differences in outcomes for patients with metastatic breast cancer by disease subtype. Breast Cancer Res Treat.

[31] Reeder-Hayes K, Hinton SP, Meng K, Carey LA, Dusetzina SB (2016). Disparities in use of human epidermal growth hormone receptor 2–targeted therapy for early-stage breast cancer. J Clin Oncol.

[32] Vaz-Luis I, Lin NU, Keating NL, Barry WT, Lii J, Burstein HJ (2016). Treatment of early-stage human epidermal growth factor 2-positive cancers among medicare enrollees: age and race strongly associated with non-use of trastuzumab. Breast Cancer Res Treat.

[33] Tsai H-T, Isaacs C, Lynce FC, O'Neill SC, Liu C, Schwartz MD (2017). Initiation of trastuzumab by women younger than 64 years for adjuvant treatment of stage I–III breast cancer. J Natl Compr Cancer Netw.

[34] Freedman RA, Hughes ME, Ottesen RA, Weeks JC, He Y, Wong Y-N (2013). Use of adjuvant trastuzumab in women with human epidermal growth factor receptor 2 (HER2)-positive breast cancer by race/ethnicity and education within the national comprehensive cancer network. Cancer..

[35] Haas JS, Phillips KA, Liang S-Y, Hassett MJ, Keohane C, Elkin EB (2011). Genomic testing and therapies for breast cancer in clinical practice. J Oncol Pract.

[36] Goldhar HA, Yan AT, Ko DT, Earle CC, Tomlinson GA, Trudeau ME, et al. The temporal risk of heart failure associated with adjuvant trastuzumab in breast cancer patients: a population study. J Natl Cancer Inst. 2015;108(1):djv301. 10.1093/jnci/djv301 26476433

[37] Kumachev A, Trudeau ME, Chan KKW (2016). Associations among socioeconomic status, patterns of care, and outcomes in breast cancer patients in a universal health care system: Ontario’s experience. Cancer..

[38] Thavendiranathan P, Abdel-Qadir H, Fischer HD, Camacho X, Amir E, Austin PC (2016). Breast cancer therapy–related cardiac dysfunction in adult women treated in routine clinical practice: a population-based cohort study. J Clin Oncol.

[39] Li J, Shao Z, Xu B, Jiang Z, Cui S, Zhang J (2018). Use of trastuzumab as an adjuvant/neoadjuvant therapy in patients with HER2-positive breast cancer in China: the Nvwa study. Medicine (Baltimore).

[40] Shih Y-CT, Elting LS, Halpern MT (2009). Factors associated with immunotherapy use among newly diagnosed cancer patients. Med Care.

[41] Zhu J, Sharma DB, Gray SW, Chen AB, Weeks JC, Schrag D (2012). Carboplatin and paclitaxel with vs without bevacizumab in older patients with advanced non–small cell lung cancer. JAMA..

[42] Langer C, Ravelo A, Hazard SJ, Guerin A, Ionescu-Ittu R, Latremouille-Viau D (2014). Comparison of survival and hospitalization rates between Medicare patients with advanced NSCLC treated with bevacizumab–carboplatin–paclitaxel and carboplatin–paclitaxel: a retrospective cohort study. Lung Cancer.

[43] Ritzwoller DP, Carroll NM, Delate T, Hornbrook MC, Kushi L, Bowles EJA (2014). Comparative effectiveness of adjunctive bevacizumab for advanced lung cancer: the cancer research network experience. J Thorac Oncol.

[44] Menter AR, Carroll NM, Sakoda LC, Delate T, Hornbrook MC, Jain RK (2017). Effect of angiotensin system inhibitors on survival in patients receiving chemotherapy for advanced non–small-cell lung cancer. Clin Lung Cancer.

[45] Maguire FB, Morris CR, Parikh-Patel A, Cress RD, Keegan THM, Li C-S, et al. First-line systemic treatments for stage IV non-small cell lung cancer in California: patterns of care and outcomes in a real-world setting. JNCI Cancer Spectrum. 2019b;3(3):pkz020. 10.1093/jncics/pkz020 PMC7050031 32328551

[46] Verma V, Haque W, Cushman TR, Lin C, II CBS, Chang JY, et al. (2019). Racial and insurance-related disparities in delivery of immunotherapy-type compounds in the United States. J Immunother.

[47] Lairson DR, Parikh RC, Cormier JN, Chan W, Du XL (2015). Cost-utility analysis of treatments for advanced non-small cell lung cancer. Am J Pharm Benefits.

[48] Sahara K, Farooq SA, Tsilimigras DI, Merath K, Paredes AZ, Wu L (2019). Immunotherapy utilization for hepatobiliary cancer in the United States: disparities among patients with different socioeconomic status. Hepatobiliary Surg Nutr.

[49] Sanoff HK, Chang Y, Lund JL, O'Neil BH, Dusetzina SB (2016). Sorafenib effectiveness in advanced hepatocellular carcinoma. Oncologist..

[50] Parsons HM, Chu Q, Karlitz JJ, Stevens JL, Harlan LC (2017). Adoption of sorafenib for the treatment of advanced-stage hepatocellular carcinoma in oncology practices in the United States. Liver Cancer.

[51] Sarpel U, Heskel M, Spivack JH, Feferman Y, Ang C, Gany F (2018). Disparities in access to sorafenib in communities with low socioeconomic status. J Health Care Poor Underserved.

[52] Al-Qurayshi Z, Crowther JE, Hamner JB, Ducoin C, Killackey MT, Kandil E (2018). Disparities of immunotherapy utilization in patients with stage III cutaneous melanoma: a national perspective. Anticancer Res.

[53] Haque W, Verma V, Butler EB, Teh BS (2019). Racial and socioeconomic disparities in the delivery of immunotherapy for metastatic melanoma in the United States. J Immunother.

[54] Fu AZ, Tsai H-T, Marshall JL, Freedman AN, Potosky AL (2014). Utilization of bevacizumab in US elderly patients with colorectal cancer receiving chemotherapy. J Oncol Pharm Pract.

[55] Meyerhardt JA, Li L, Sanoff HK, Carpenter W, Schrag D (2012). Effectiveness of bevacizumab with first-line combination chemotherapy for Medicare patients with stage IV colorectal cancer. J Clin Oncol.

[56] Cen P, Liu C, Du XL (2012). Comparison of toxicity profiles of fluorouracil versus oxaliplatin regimens in a large population-based cohort of elderly patients with colorectal cancer. Ann Oncol.

[57] Parikh RC, Du XL, Morgan RO, Lairson DR (2016). Patterns of treatment sequences in chemotherapy and targeted biologics for metastatic colorectal cancer: findings from a large community-based cohort of elderly patients. Drugs Real World Outcomes.

[58] Neugut AI, Becker DJ, Insel BJ, Hershman DL (2012). Uptake of oxaliplatin and bevacizumab for treatment of node-positive and metastatic colon cancer. J Oncol Pract.

[59] Raab GT, Lin A, Hillyer GC, Keller D, O’Neil DS, Accordino MK (2019). Use of bevacizumab for elderly patients with stage IV colon cancer: analysis of SEER-Medicare data. Clin Colorectal Cancer.

[60] Taylor JP, Stem M, Yu D, Chen SY, Fang SH, Gearhart SL (2019). Treatment strategies and survival trends for anorectal melanoma: is it time for a change?. World J Surgery.

[61] Saigal CS, Deibert CM, Lai J, Schonlau M (2010). Disparities in the treatment of patients with IL-2 for metastatic renal cell carcinoma. Urol Oncol.

[62] Amini A, Eguchi M, Jones BL, Stokes WA, Gupta A, McDermott JD (2018). Comparing outcomes of concurrent chemotherapy regimens in patients 65 years old or older with locally advanced oropharyngeal carcinoma. Cancer..

[63] Xiang M, Holsinger FC, Colevas AD, Chen MM, Le Q-T, Beadle BM (2018). Survival of patients with head and neck cancer treated with definitive radiotherapy and concurrent cisplatin or concurrent cetuximab: a Surveillance, Epidemiology, and End Results-Medicare analysis. Cancer..

[64] Hershman DL, Wright JD, Lim E, Buono DL, Tsai WY, Neugut AI (2013). Contraindicated use of bevacizumab and toxicity in elderly patients with cancer. J Clin Oncol.

[65] Mohile SG, Hardt M, Tew W, Owusu C, Klepin H, Gross C (2013). Toxicity of bevacizumab in combination with chemotherapy in older patients. Oncologist..

[66] Enewold L, Sharon E, Harlan LC (2017). Metastatic melanoma: treatment and survival in the US after the introduction of ipilimumab and vemurafenib. Oncology Res Treat.

[67] Enewold L, Thomas A (2016). Real-world patterns of EGFR testing and treatment with erlotinib for non-small cell lung cancer in the United States. PLoS One.

[68] Palazzo LL, Sheehan DF, Tramontano AC, Kong CY (2019). Disparities and trends in genetic testing and erlotinib treatment among metastatic non–small cell lung cancer patients. Cancer Epidemiology Biomarkers Prev..

[69] Freedman RA, Vaz-Luis I, Barry WT, Lii H, Lin NU, Winer EP (2014). Patterns of chemotherapy, toxicity, and short-term outcomes for older women receiving adjuvant trastuzumab-based therapy. Breast Cancer Res Treat.

[70] Vaz-Luis I, Keating NL, Lin NU, Lii H, Winer EP, Freedman RA (2014). Duration and toxicity of adjuvant trastuzumab in older patients with early-stage breast cancer: a population-based study. J Clin Oncol.

[71] Reeder-Hayes KE, Meyer AM, Hinton SP, Meng K, Carey LA, Dusetzina SB (2017). Comparative toxicity and effectiveness of trastuzumab-based chemotherapy regimens in older women with early-stage breast cancer. J Clinic Oncol.

[72] Chavez-MacGregor M, Niu J, Zhang N, Elting LS, Smith BD, Banchs J (2015). Cardiac monitoring during adjuvant trastuzumab-based chemotherapy among older patients with breast cancer. J Clin Oncol.

[73] Lu CY, Srasuebkul P, Drew AK, Chen K, Ward RL, Pearson S-A (2013). Trastuzumab therapy in Australia: which patients with HER2+ metastatic breast cancer are assessed for cardiac function?. Breast..

[74] Krimphove MJ, Tully KH, Friedlander DF, Marchese M, Ravi P, Lipsitz SR (2019). Adoption of immunotherapy in the community for patients diagnosed with metastatic melanoma. J Immunother Cancer.

[75] Zheng Z, Hanna N, Onukwugha E, Reese ES, Seal B, Mullins CD (2014). Does the type of first-line regimens influence the receipt of second-line chemotherapy treatment? An analysis of 3,211 metastatic colon cancer patients. Cancer Med.

[76] Kwan SW, Harris WP, Gold LS, Hebert PL (2018). Comparative effectiveness of transarterial embolization and sorafenib for hepatocellular carcinoma: a population-based study. Am J Roentgenol.

[77] Li P, Jahnke J, Pettit AR, Wong Y-N, Doshi JA (2019). Comparative survival associated with use of targeted vs nontargeted therapy in medicare patients with metastatic renal cell carcinoma. JAMA Netw Open.

[78] Zandberg DP, Cullen K, Bentzen SM, Goloubeva OG (2018). Definitive radiation with concurrent cetuximab vs. radiation with or without concurrent cytotoxic chemotherapy in older patients with squamous cell carcinoma of the head and neck: analysis of the SEER-medicare linked database. Oral Oncol.

[79] Thavendiranathan P, Abdel-Qadir H, Fischer HD, Liu Y, Camacho X, Amir E (2018). Risk-imaging mismatch in cardiac imaging practices for women receiving systemic therapy for early-stage breast cancer: a population-based cohort study. J Clin Oncol.

[80] Delate T, Won K, Nm C, Kushi L, Hornbrook M, Eja B (2014). Factors associated with first-line bevacizumab use in advanced non-squamous non-small cell lung cancer. J Cancer Res Ther (Manch).

[81] Carroll NM, Delate T, Menter A, Hornbrook MC, Kushi L, Bowles EJA (2015). Use of bevacizumab in community settings: toxicity profile and risk of hospitalization in patients with advanced non–small-cell lung cancer. J Oncol Pract..

[82] Maguire FB, Morris CR, Parikh-Patel A, Cress RD, Keegan THM, Li C-S (2019). Disparities in systemic treatment use in advanced-stage non–small cell lung cancer by source of health insurance. Cancer Epidemiol Biomarkers Prev.

[83] Smith D, Thomson K, Bambra C, Todd A (2019). The breast cancer paradox: a systematic review of the association between area-level deprivation and breast cancer screening uptake in Europe. Cancer Epidemiol.

[84] Lyratzopoulos G, Abel GA, Brown CH, Rous BA, Vernon SA, Roland M (2013). Socio-demographic inequalities in stage of cancer diagnosis: evidence from patients with female breast, lung, colon, rectal, prostate, renal, bladder, melanoma, ovarian and endometrial cancer. Ann Oncol.

[85] Hayes L AJ, McCallum I, Forrest L, Hidajat M, White M, Sharp L. Age-related and socio-economic inequalities in timeliness of referral and start of treatment in colorectal cancer. A population-based analysis J Epi Comm Health 2020; in press. 10.1136/jech-2020-214232 33055178

[86] Woods LM, Rachet B, Coleman MP (2006). Origins of socio-economic inequalities in cancer survival: a review. Ann Oncol.

[87] Network NCI. Evidence to March 2010 on cancer inequalities in England. 2010. www.ncin.org.uk/inequalities. Accessed 17 Mar 2020.

[88] Sarfati D, Koczwara B, Jackson C (2016). The impact of comorbidity on cancer and its treatment. CA Cancer J Clin.

[89] Dahlgren G, Whitehead M. Tackling inequalities in health: what can we learn from what has been tried? Working paper prepared for the King’s Fund international seminar on tackling inequalities in health, September 1993, Ditchley Park, Oxfordshire. London, King’s Fund. In: Dahlgren G, Whitehead M, editors. European strategies for tackling social inequities in health: Levelling up part 2 Copenhagen: WHO Regional Office for Europe 2007. p. 20–32.

[90] Martin AP, Downing J, Cochrane M, Collins B, Francis B, Haycox A (2018). Trastuzumab uptake in HER2-positive breast cancer patients: a systematic review and meta-analysis of observational studies. Crit Rev Oncol/Hematol.

[91] Osbourne CK (1998). Steroid hormone receptors in breast cancer management. Breast Cancer Res Treat.

[92] Victora CG, Vaughan JP, Barros FC, Silva AC, Tomasi E (2000). Explaining trends in inequities: evidence from Brazilian child health studies. Lancet..

[93] Rodgers E (2003). Diffusion of innovations.

[94] White M, Adams J, Heywood P, Babones SJ (2012). How and why do interventions that increase health overall widen inequalities within populations?. Social inequality and public health.

[95] Tseng C-H, Chiang C-J, Tseng J-S, Yang T-Y, Hsu K-H, Chen K-C (2017). EGFR mutation, smoking, and gender in advanced lung adenocarcinoma. Oncotarget..

[96] Lung Cancer Coalition. Molecules matter turning the science of molecular diagnostics in lung cancer into a practical service for all patients. 2019. https://www.uklcc.org.uk/wp-content/uploads/2019/01/Molecules-Matter.pdf. Accessed 15 Mar 2020.

[97] Lee DH, Tsao M-S, Kambartel K-O, Isobe H, Huang M-S, Barrios CH (2018). Molecular testing and treatment patterns for patients with advanced non-small cell lung cancer: PIvOTAL observational study. PloS One.

[98] Illei PB, Wong W, Wu N, Chu L, Gupta R, Schulze K (2018). ALK testing trends and patterns among community practices in the United States. JCO Precis Oncol.

[99] Cancer Research UK. Cancer Research UK policy statement: patient access to molecular diagnostics and targeted medicines in England. 2018. https://www.cancerresearchuk.org/sites/default/files/access_to_molecular_diagnostic_tests_and_targeted_medicines_in_england_0.pdf. Accessed 15 Mar 2020.

[100] Deeks JJ, Higgins JPT, Altman DG. Chapter 10: analysing data and undertaking meta-analyses, Section 4.6.4 In: Higgins JPT, Green S, editors. Cochrane handbook for systematic reviews of interventions. Version 510 [updated March 2011]. Chichester: Wiley; 2011.

[101] Galobardes B, Shaw M, Lawlor DA, Lynch JW, Davey SG (2006). Indicators of socioeconomic position (part 1). Journal Epidemiol Community Health.

[102] Bach PB, Guadagnoli E, Schrag D, Schussler N, Warren JL (2002). Patient demographic and socioeconomic characteristics in the SEER-Medicare database: applications and limitations. Med Care.

[103] Griggs JJ (2012). Role of nonclinical factors in the receipt of high-quality systemic adjuvant breast cancer treatment. J Clinical Oncol.

[104] Goss E, Lopez AM, Brown CL, Wollins DS, Brawley OW, Raghavan D (2009). American Society of Clinical Oncology policy statement: disparities in cancer care. J Clin Oncol.

