# Supplementary material for: Are there socio-economic inequalities in utilization of predictive biomarker tests and biological and precision therapies for cancer? A systematic review and meta-analysis
Source: BMC Med. 2020 Oct 23;18:282. doi: 10.1186/s12916-020-01753-0 (PMC7583194; doi:10.1186/s12916-020-01753-0)
Supplement: Supplementary file 1 — Additional file 1: Supplementary methods 1, PRISMA checklist. Supplementary methods 2, search strategy. Supplementary methods 3, inclusion/exclusion decision trees. Supplementary methods 4, quality appraisal tool. Table S1, no denominator/mean socio-economic status only study characteristics. Fig. S1, SEER versus non-SEER registry studies forest plot and funnel plot. Table S2, included studies characteristics. Table S3, quality appraisal results breakdown. Fig. S2, sensitivity analyses. Fig. S3, all other cancers forest plot and funnel plot. Fig. S4, breast, lung and all other cancers forest plot and funnel plot. Fig. S5, additional funnel plots. [file 12916_2020_1753_MOESM1_ESM.docx]

**Additional File 1**

**Are there socio-economic inequalities in utilization of predictive biomarker tests and biological and precision therapies for cancer? A systematic review and meta-analysis**

Norris. R. P^1,2^, Dew. R^2^, Sharp. L^2^, Greystoke. A^3^, Rice. S^4^, Johnell. K^5^. and Todd. A^1,2*^

^1^School of Pharmacy, Newcastle University, Newcastle-upon-Tyne, United Kingdom.

^2^Population Health Sciences Institute, Newcastle University Centre for Cancer, Newcastle-upon-Tyne, United Kingdom.

^3^Newcastle University Centre for Cancer, Newcastle-upon-Tyne, United Kingdom.

^4^Health Economics Group and Evidence Synthesis Team, Population Health Sciences Institute, Newcastle University, Newcastle-upon-Tyne, United Kingdom.

^5^Department of Medical Epidemiology and Biostatistics, Karolinska Institutet, Stockholm, Sweden.

*Corresponding author: [adam.todd@newcastle.ac.uk](mailto:adam.todd@newcastle.ac.uk)

**Table of Contents**

| **Supplementary Methods 1:** PRISMA checklist………………………………………… | 3 |
| --- | --- |
| **Supplementary Methods 2:** Search strategy…………………………………………….. | 6 |
| **Supplementary Methods 3:** Inclusion/exclusion decision trees…………………………. | 20 |
| **Supplementary Methods 4:** Quality appraisal tool……………………………………… | 22 |
| **Table S1:** No denominator/mean socio-economic status only studies characteristics……. | 23 |
| **Figure S1:** SEER versus non-SEER registry studies forest plot and funnel plot………… | 27 |
| **Table S2:** Included studies characteristics………………………………………………... | 29 |
| **Table S3:** Quality appraisal results breakdown…………………………………………... | 46 |
| **Figure S2:** Sensitivity analyses…………………………………………………………… | 48 |
| **Figure S3:** All other cancers forest plot and funnel plot………………………………….. | 50 |
| **Figure S4:** Breast, lung and all other cancers forest plot and funnel plot………………… | 52 |
| **Figure S5:** Additional funnel plots……………………………………………………….. | 54 |

**Supplementary Methods 1: PRISMA Checklist**

| **Section/topic** | **#** | **Checklist item** | **Reported on page #** |  |
| --- | --- | --- | --- | --- |
| **TITLE** | | |  |  |
| Title | 1 | Identify the report as a systematic review, meta-analysis, or both. | 1 |  |
| **ABSTRACT** | | |  |  |
| Structured summary | 2 | Provide a structured summary including, as applicable: background; objectives; data sources; study eligibility criteria, participants, and interventions; study appraisal and synthesis methods; results; limitations; conclusions and implications of key findings; systematic review registration number. | 2 & 3 |  |
| **INTRODUCTION** | | |  |  |
| Rationale | 3 | Describe the rationale for the review in the context of what is already known. | 4 |  |
| Objectives | 4 | Provide an explicit statement of questions being addressed with reference to participants, interventions, comparisons, outcomes, and study design (PICOS). | 4 & 5 |  |
| **METHODS** | | |  |  |
| Protocol and registration | 5 | Indicate if a review protocol exists, if and where it can be accessed (e.g., Web address), and, if available, provide registration information including registration number. | NA |  |
| Eligibility criteria | 6 | Specify study characteristics (e.g., PICOS, length of follow-up) and report characteristics (e.g., years considered, language, publication status) used as criteria for eligibility, giving rationale. | 5 |  |
| Information sources | 7 | Describe all information sources (e.g., databases with dates of coverage, contact with study authors to identify additional studies) in the search and date last searched. | 5 |  |
| Search | 8 | Present full electronic search strategy for at least one database, including any limits used, such that it could be repeated. | 5,  Supp Methods 2 |  |
| Study selection | 9 | State the process for selecting studies (i.e., screening, eligibility, included in systematic review, and, if applicable, included in the meta-analysis). | 5, 6, 7 &  Supp Methods 3 |  |
| Data collection process | 10 | Describe method of data extraction from reports (e.g., piloted forms, independently, in duplicate) and any processes for obtaining and confirming data from investigators. | 6 |  |
| Data items | 11 | List and define all variables for which data were sought (e.g., PICOS, funding sources) and any assumptions and simplifications made. | 6 & 7 |  |
| Section/topic | # | Checklist item | Reported on page # |  |
| Risk of bias in individual studies | 12 | Describe methods used for assessing risk of bias of individual studies (including specification of whether this was done at the study or outcome level), and how this information is to be used in any data synthesis. | 6 & 7 |  |
| Summary measures | 13 | State the principal summary measures (e.g., risk ratio, difference in means). | 7 |  |
| Synthesis of results | 14 | Describe the methods of handling data and combining results of studies, if done, including measures of consistency (e.g., I2) for each meta-analysis. | 7 & 8 |  |
| Risk of bias across studies | 15 | Specify any assessment of risk of bias that may affect the cumulative evidence (e.g., publication bias, selective reporting within studies). | 7 & 8 &  Supp Methods 4 |  |
| Additional analyses | 16 | Describe methods of additional analyses (e.g., sensitivity or subgroup analyses, meta-regression), if done, indicating which were pre-specified. | 7 & 8 |  |
| **RESULTS** | | | |  |
| Study selection | | 17 | Give numbers of studies screened, assessed for eligibility, and included in the review, with reasons for exclusions at each stage, ideally with a flow diagram. | 8 & Figure 1 |
| Study characteristics | | 18 | For each study, present characteristics for which data were extracted (e.g., study size, PICOS, follow-up period) and provide the citations. | 8, 9, 10 & 11 &  Tables S1 & S2 |
| Risk of bias within studies | | 19 | Present data on risk of bias of each study and, if available, any outcome level assessment (see item 12). | 9 & Table S3 |
| Results of individual studies | | 20 | For all outcomes considered (benefits or harms), present, for each study: (a) simple summary data for each intervention group (b) effect estimates and confidence intervals, ideally with a forest plot. | 8, 9,10 & 11 Figures S1 & S3,  Figures 2, 3, 4 & 5 |
| Synthesis of results | | 21 | Present results of each meta-analysis done, including confidence intervals and measures of consistency. | 8, 9, & 10, Figures S1 & S3,  Figures 2, 3, 4 & 5 |
| Risk of bias across studies | | 22 | Present results of any assessment of risk of bias across studies (see Item 15). | 9 & Table S3 |

| **Section/topic** | **#** | **Checklist item** | **Reported on page #** |
| --- | --- | --- | --- |
| Additional analysis | 23 | Give results of additional analyses, if done (e.g., sensitivity or subgroup analyses, meta-regression [see Item 16]). | 10 & 11  Figure S2 & S4 |
| **DISCUSSION** | | |  |
| Summary of evidence | 24 | Summarize the main findings including the strength of evidence for each main outcome; consider their relevance to key groups (e.g., healthcare providers, users, and policy makers). | 11, 12, 13, 14 & 15 |
| Limitations | 25 | Discuss limitations at study and outcome level (e.g., risk of bias), and at review-level (e.g., incomplete retrieval of identified research, reporting bias). | 13 & 14 |
| Conclusions | 26 | Provide a general interpretation of the results in the context of other evidence, and implications for future research. | 11, 12, 13, 14 & 15 |
| **FUNDING** | | |  |
| Funding | 27 | Describe sources of funding for the systematic review and other support (e.g., supply of data); role of funders for the systematic review. | 16 |

*From:*  Moher D, Liberati A, Tetzlaff J, Altman DG, The PRISMA Group (2009). Preferred Reporting Items for Systematic Reviews and Meta-Analyses: The PRISMA Statement. PLoS Med 6(7): e1000097. doi:10.1371/journal.pmed1000097

**Supplementary Methods 2: Database search strategy**

**A. Medline search strategy 04/02/2019 (kept updated through to 31/12/2019)**

|  | **Search Term** | **Number Retrieved** |
| --- | --- | --- |
| 1. | alectinib.mp | 218 |
| 2. | ceritinib.mp. | 250 |
| 3. | Crizotinib/ | 948 |
| 4. | crizotinib.mp. | 1,426 |
| 5. | brigatinib.mp. | 35 |
| 6. | Erlotinib Hydrochloride/ | 3,524 |
| 7. | erlotinib.mp. | 5,180 |
| 8. | Gefitinib/ | 4,090 |
| 9. | gefitinib.mp. | 5,613 |
| 10. | Afatinib/ | 482 |
| 11. | afatinib.mp. | 736 |
| 12. | osimertinib.mp. | 231 |
| 13. | Imatinib Mesylate/ | 9,511 |
| 14. | imatinib.mp. | 12,608 |
| 15. | Lapatinib/ | 1,407 |
| 16. | lapatinib.mp. | 2,064 |
| 17. | neratinib.mp. | 137 |
| 18. | masitinib.mp. | 101 |
| 19. | lenvatinib.mp. | 227 |
| 20. | cabozantinib.mp. | 412 |
| 21. | Sunitinib/ | 3,150 |
| 22. | sunitinib.mp. | 4,567 |
| 23. | Axitinib/ | 406 |
| 24. | axitinib.mp. | 637 |
| 25. | pazopanib.mp. | 1,095 |
| 26. | tivozanib.mp. | 71 |
| 27. | vandetanib.mp. | 516 |
| 28. | nintedanib.mp. | 428 |
| 29. | regorafenib.mp. | 516 |
| 30. | Sorafenib/ | 4,170 |
| 31. | sorafenib.mp. | 6,010 |
| 32. | dabrafenib.mp. | 614 |
| 33. | Vemurafenib/ | 1,085 |
| 34. | vemurafenib.mp. | 1,553 |
| 35. | encorafenib.mp. | 11 |
| 36. | cobimetinib.mp. | 91 |
| 37. | trametinib.mp. | 581 |
| 38. | binimetinib.mp. | 35 |
| 39. | Everolimus/ | 4,079 |
| 40. | everolimus.mp. | 5,388 |
| 41. | temsirolimus.mp. | 1,239 |
| 42. | abemaciclib.mp. | 75 |
| 43. | palbociclib.mp. | 385 |
| 44. | ribociclib.mp. | 101 |
| 45. | niraparib.mp. | 63 |
| 46. | olaparib.mp. | 689 |
| 47. | rucaparib.mp. | 118 |
| 48. | vismodegib.mp. | 356 |
| 49. | sonidegib.mp. | 62 |
| 50. | Cetuximab/ | 3,976 |
| 51. | cetuximab.mp. | 5,544 |
| 52. | Panitumumab/ | 851 |
| 53. | panitumumab.mp. | 1,327 |
| 54. | pertuzumab.mp. | 647 |
| 55. | Trastuzumab/ | 6,015 |
| 56. | trastuzumab.mp. | 8,416 |
| 57. | trastuzumab emtansine.mp. | 386 |
| 58. | exp Tamoxifen/ | 15,087 |
| 59. | tamoxifen.mp. | 18,378 |
| 60. | Fulvestrant/ | 2,080 |
| 61. | fulvestrant.mp. | 2,510 |
| 62. | toremifene.mp. | 520 |
| 63. | Megestrol Acetate/ | 512 |
| 64. | megestrol acetate.mp. | 771 |
| 65. | Medroxyprogesterone Acetate/ | 2,936 |
| 66. | medroxyprogesterone acetate.mp. | 3,923 |
| 67. | Anastrozole/ | 1,301 |
| 68. | anastrozole.mp. | 1,861 |
| 69. | exemestane.mp. | 1,151 |
| 70. | Letrozole/ | 1,787 |
| 71. | letrozole.mp. | 2,471 |
| 72. | Goserelin/ | 1,020 |
| 73. | goserelin.mp. | 1,182 |
| 74. | Buserelin/ | 644 |
| 75. | buserelin.mp. | 789 |
| 76. | leuprorelin acetate.mp. | 185 |
| 77. | Leuprolide/ | 2,010 |
| 78. | leuprolide.mp. | 2,297 |
| 79. | Triptorelin Pamoate/ | 1,098 |
| 80. | triptorelin pamoate.mp. | 1,100 |
| 81. | triptorelin.mp. | 1,228 |
| 82. | anamorelin.mp. | 29 |
| 83. | Abiraterone Acetate/ | 305 |
| 84. | abiraterone acetate.mp. | 602 |
| 85. | bicalutamide.mp. | 1,399 |
| 86. | enzalutamide.mp. | 846 |
| 87. | Flutamide/ | 1,767 |
| 88. | flutamide.mp. | 2,452 |
| 89. | Diethylstilbestrol/ | 1,744 |
| 90. | diethylstilbestrol.mp. | 2,553 |
| 91. | interleukin.mp. | 246,595 |
| 92. | mifamurtide.mp. | 61 |
| 93. | talimogene laherparepvec.mp. | 75 |
| 94. | avelumab.mp. | 49 |
| 95. | Nivolumab/ | 1,155 |
| 96. | nivolumab.mp. | 1,645 |
| 97. | durvalumab.mp. | 100 |
| 98. | Ipilimumab/ | 1,354 |
| 99. | ipilimumab.mp. | 1,937 |
| 100. | necitumumab.mp. | 52 |
| 101. | pembrolizumab.mp. | 1,163 |
| 102. | atezolizumab.mp. | 281 |
| 103. | dinutuximab.mp. | 25 |
| 104. | olaratumab.mp. | 36 |
| 105. | Bevacizumab/ | 10,270 |
| 106. | bevacizumab.mp. | 13,381 |
| 107. | ramucirumab.mp. | 355 |
| 108. | aflibercept.mp. | 1,233 |
| 109. | aldesleukin.mp. | 111 |
| 110. | degarelix.mp. | 150 |
| 111. | Protein Kinase Inhibitors/ | 37,421 |
| 112. | protein kinase inhibitor*.mp. | 39,698 |
| 113. | tyrosine kinase inhibitor*.mp. | 19,290 |
| 114. | tyrosine protein kinase inhibitor*.mp. | 41 |
| 115. | TKI.mp. | 4,410 |
| 116. | TKIs.mp. | 3,897 |
| 117. | multireceptor tyrosine kinase inhibitor*.mp. | 6 |
| 118. | BRAF kinase inhibitor*.mp. | 52 |
| 119. | MAPK inhibitor*.mp. | 3,426 |
| 120. | mitogen-activated protein kinase inhibitor*.mp. | 541 |
| 121. | MEK inhibitor*.mp. | 4,070 |
| 122. | mTOR inhibitor*.mp. | 4,675 |
| 123. | CDK inhibitor*.mp. | 3,450 |
| 124. | Cyclin dependent kinase inhibitor*.mp. | 25,818 |
| 125. | “Poly(ADP-ribose) Polymerase Inhibitors"/ | 2,727 |
| 126. | "Poly(ADP-ribose) Polymerase Inhibitors".mp. | 2,822 |
| 127. | PARP inhibitor*.mp. | 2,055 |
| 128. | Hedgehog pathway inhibitor*.mp. | 180 |
| 129. | Interleukins/ | 12,436 |
| 130. | Interleukins.mp. | 15,799 |
| 131. | Anti-oestrogen*.mp. | 408 |
| 132. | Anti-estrogen*.mp. | 1,685 |
| 133. | Progesterone/ | 21,305 |
| 134. | Progesterone.mp. | 50,516 |
| 135. | Aromatase Inhibitors/ | 5,042 |
| 136. | Aromatase inhibitor*.mp. | 6,875 |
| 137. | Gonadotropin-Releasing Hormone/ | 13,746 |
| 138. | Gonadotropin-releasing hormone.mp. | 16,400 |
| 139. | Androgen Antagonists/ | 8,314 |
| 140. | androgen antagonists.mp. | 8,333 |
| 141. | Anti-androgen*.mp. | 1,848 |
| 142. | Androgen Receptor Antagonists/ | 1,096 |
| 143. | Androgen receptor antagonists.mp. | 1,165 |
| 144. | Estrogens/ | 25,434 |
| 145. | estrogen*.mp. | 109,215 |
| 146. | oestrogen*.mp. | 10,504 |
| 147. | Anti-gonadotrophin releasing hormone*.mp. | 3 |
| 148. | VEGF inhibitor*.mp. | 676 |
| 149. | Vascular endothelial growth factor inhibitor*.mp. | 235 |
| 150. | Angiogenesis Inhibitors/ | 22,825 |
| 151. | Angiogenesis inhibitor*.mp. | 24,103 |
| 152. | EGFR inhibitor*.mp. | 2,805 |
| 153. | Epidermal growth factor inhibitor*.mp. | 51 |
| 154. | ALK inhibitor*.mp. | 761 |
| 155. | cMET inhibitor*.mp. | 18 |
| 156. | Antibodies, Monoclonal/ | 107,607 |
| 157. | Monoclonal antibod*.mp. | 91,652 |
| 158. | MAB*.mp. | 33,975 |
| 159. | exp Leukemia/ | 108,260 |
| 160. | leukemia.mp. | 150,191 |
| 161. | leukaemia.mp. | 18,017 |
| 162. | exp Lymphoma/ | 85,842 |
| 163. | lymphoma.mp. | 130,464 |
| 164. | exp Arthritis, Rheumatoid/ | 56,908 |
| 165. | Rheumatoid arthritis.mp. | 58,515 |
| 166. | exp Inflammatory Bowel Diseases/ | 50,019 |
| 167. | Inflammatory bowel disease*.mp. | 34,931 |
| 168. | ulcerative colitis.mp. | 21,823 |
| 169. | Crohn* disease.mp. | 31,880 |
| 170. | exp Psoriasis/ | 21,775 |
| 171. | Psoriasis.mp. | 24,020 |
| 172. | Spondylitis, Ankylosing/ | 7,001 |
| 173. | Ankylosing spondylitis.mp. | 7,715 |
| 174. | (Or/159 – 173) [OR for MAB uses that we are not interested in] | 412,207 |
| 175. | 156 or 157 or 158 [MAB synonyms] | 159,349 |
| 176. | 175 not 174 [MABs but not disease states we are not interested in] | 134,547 |
| 177. | targeted treatment*.mp. | 3,876 |
| 178. | targeted technologies.mp. | 10 |
| 179. | personalized treatment.mp. | 1,770 |
| 180. | personalised treatment.mp. | 173 |
| 181. | targeted therapy.mp. | 35,555 |
| 182. | targeted therapies.mp. | 13,546 |
| 183. | Precision Medicine/ | 13,508 |
| 184. | precision medicine.mp. | 15,115 |
| 185. | molecularly targeted drug*.mp. | 201 |
| 186. | Molecular Targeted Therapy/ | 23,285 |
| 187. | Molecular targeted therap*.mp. | 24,488 |
| 188. | Personalised medicine.mp. | 666 |
| 189. | Personalized medicine.mp. | 6,925 |
| 190. | Tailored medicine.mp. | 53 |
| 191. | Genomic medicine.mp. | 800 |
| 192. | Precision cancer care.mp. | 12 |
| 193. | Stratified medicine.mp. | 194 |
| 194. | (molecular adj2 test).mp. | 1,141 |
| 195. | genomic testing.mp. | 390 |
| 196. | targeted test*.mp. | 254 |
| 197. | biomarker test*.mp. | 520 |
| 198. | molecular test*.mp. | 4,224 |
| 199. | mutation* test*.mp. | 1,635 |
| 200. | test* trend*.mp. | 118 |
| 201. | test* pattern.mp.1 | 257 |
| 202. | genetic profile/ | 10 |
| 203. | genetic profile.mp. | 1,537 |
| 204. | Genetic Testing/ | 31,258 |
| 205. | genetic testing.mp. | 38,351 |
| 206. | Pharmacogenomic Testing/ | 341 |
| 207. | Pharmacogenomic testing.mp. | 491 |
| 208. | Pharmacogenetic testing.mp. | 438 |
| 209. | molecular profil*.mp. | 4,521 |
| 210. | molecular diagnostics.mp. | 3,158 |
| 211. | genotyping.mp. | 47,260 |
| 212. | Genomic profiling.mp. | 1,132 |
| 213. | Gene test*.mp. | 1,000 |
| 214. | Molecular Diagnostic Techniques/ | 9,673 |
| 215. | Molecular diagnostic techniques.mp. | 9,937 |
| 216. | (EGFR adj2 test*).mp. | 502 |
| 217. | HER2 test*.mp. | 426 |
| 218. | ALK test*.mp. | 68 |
| 219. | BRAF test*.mp. | 69 |
| 220. | ER test*.mp. | 35 |
| 221. | PR test*.mp. | 63 |
| 222. | KIT test*.mp. | 81 |
| 223. | BRCA test*.mp. | 241 |
| 224. | RET test*.mp. | 18 |
| 225. | MET test*.mp. | 26 |
| 226. | KRAS test*.mp. | 121 |
| 227. | NRAS test*.mp. | 4 |
| 228. | PD1 test*.mp. | 1 |
| 229. | CTLA4 test*.mp. | 1 |
| 230. | Receipt of.mp. | 12,742 |
| 231. | treatment with.mp. | 316,651 |
| 232. | receive*.mp. | 670,956 |
| 233. | non-use.mp. | 1,743 |
| 234. | treatment receipt.mp. | 98 |
| 235. | receipt.mp. | 12,748 |
| 236. | initiat*.mp. | 393,392 |
| 237. | treatment utilisation.mp. | 38 |
| 238. | Treatment utilization.mp. | 526 |
| 239. | utili#ation.mp. | 129,587 |
| 240. | Access.mp. | 202,172 |
| 241. | Underutilisation.mp. | 128 |
| 242. | Underutilization.mp. | 1,314 |
| 243. | Treatment barrier*.mp. | 313 |
| 244. | Non receipt.mp. | 66 |
| 245. | Non-initiation.mp. | 51 |
| 246. | Treatment pattern*.mp. | 2,690 |
| 247. | Utilization pattern*.mp. | 1,684 |
| 248. | Utilisation pattern*.mp. | 176 |
| 249. | Drug Utilization/ | 14,097 |
| 250. | Drug utilisation.mp. | 279 |
| 251. | Drug Utilization.mp. | 17,809 |
| 252. | Uptake.mp. | 219,854 |
| 253. | Provision.mp. | 48,132 |
| 254. | (Or/230 – 253) [Utilization synonyms OR] | 1,805,579 |
| 255. | Socioeconomic status.mp. | 25,641 |
| 256. | exp Socioeconomic Factors/ | 284,680 |
| 257. | Socioeconomic factor*.mp. | 107,585 |
| 258. | Poverty.mp. | 40,752 |
| 259. | Poverty areas.mp. | 5,013 |
| 260. | Social class.mp. | 27,739 |
| 261. | Social mobility.mp. | 747 |
| 262. | Index of multiple deprivation.mp. | 401 |
| 263. | Socioeconomic position.mp. | 2,187 |
| 264. | Carstairs index.mp. | 40 |
| 265. | Townsend Index.mp. | 73 |
| 266. | Area level deprivation.mp. | 117 |
| 267. | Poverty level.mp. | 1,122 |
| 268. | Income level.mp. | 1,570 |
| 269. | (Income adj6 category).mp. | 237 |
| 270. | Household income.mp. | 6,034 |
| 271. | (Median adj2 income).mp. | 1,254 |
| 272. | Education status.mp. | 400 |
| 273. | Education adj6 demographic).mp. | 1,443 |
| 274. | (Education adj6 variable).mp. | 343 |
| 275. | Education level.mp. | 9,065 |
| 276. | Employment status.mp. | 4,675 |
| 277. | Employment characteristic*.mp. | 162 |
| 278. | (Employment adj6 variable).mp. | 81 |
| 279. | Unemployed.mp. | 5,376 |
| 280. | Unemploy* adj6 variable).mp. | 30 |
| 281. | (Disab* adj6 variable).mp. | 380 |
| 282. | Race ethnicity.mp. | 13,358 |
| 283. | (Race adj6 variable).mp. | 287 |
| 284. | (Ethnicity adj6 variable).mp. | 169 |
| 285. | Smokers/ | 594 |
| 286. | Smoker*.mp. | 57,786 |
| 287. | Non-Smokers/ | 20 |
| 288. | Non-smoker*.mp. | 9,973 |
| 289. | Smoking adj2 history).mp. | 8,853 |
| 290. | health insurance status.mp. | 652 |
| 291. | socioeconomic.mp. | 139,337 |
| 292. | exp Medicare/ | 29,191 |
| 293. | Medicare.mp. | 42,291 |
| 294. | Race.ti,ab. | 68,527 |
| 295. | Medicaid/ | 15,589 |
| 296. | medicaid.mp. | 29,827 |
| 297. | exp Insurance Coverage/ | 15,004 |
| 298. | insurance coverage.mp. | 15,415 |
| 299. | health plan type.mp. | 35 |
| 300. | public insurance.mp. | 950 |
| 301. | Medically Uninsured/ | 6,285 |
| 302. | Medically uninsured.mp. | 6,300 |
| 303. | insurance adj6 variable).mp. | 92 |
| 304. | Commercial insurance.mp. | 490 |
| 305. | Private insurance.mp. | 2,878 |
| 306. | Military insurance.mp. | 11 |
| 307. | Other insurance.mp. | 154 |
| 308. | Racial.ti,ab. | 26,166 |
| 309. | Socioeconomics.mp. | 363 |
| 310. | (Or/1 – 155) or (Or/176 - 193) [Targeted therapy OR] | 729,966 |
| 311. | (Or/194 – 299) [Molecular Testing OR] | 109,759 |
| 312. | Or/255 – 309) [Socio-economic status OR] | 509,341 |
| 313. | 310 or 311 [Targeted therapy or molecular testing] | 829,983 |
| 314. | 313 and 254 and 312 [Targeted therapy or molecular testing - and utilization  and socio-economic status] | 2,370 |
| 315. | limit 314 to yr="1998 -Current" | 2,308 |

**B. Embase search strategy 04/02/2019 (kept updated through to 31/12/2019)**

|  | **Search term** | **Number Retrieved** |
| --- | --- | --- |
| 1. | alectinib/ | 1,139 |
| 2. | Alectinib.mp. | 1,186 |
| 3. | ceritinib/ | 1,309 |
| 4. | Ceritinib.mp. | 1,371 |
| 5. | crizotinib/ | 6,436 |
| 6. | Crizotinib.mp. | 6,748 |
| 7. | brigatinib/ | 437 |
| 8. | Brigatinib.mp. | 446 |
| 9. | erlotinib/ | 24,988 |
| 10 | Erlotinib.mp. | 25,749 |
| 11. | gefitinib/ | 22,545 |
| 12. | Gefitinib.mp. | 23,272 |
| 13. | afatinib/ | 4,087 |
| 14. | Afatinib.mp. | 4,227 |
| 15. | osimertinib/ | 1,822 |
| 16. | Osimertinib.mp. | 1,892 |
| 17. | imatinib/ | 39,035 |
| 18. | Imatinib.mp. | 40,550 |
| 19. | lapatinib/ | 10,951 |
| 20. | Lapatinib.mp. | 11,245 |
| 21. | neratinib/ | 1,254 |
| 22. | Neratinib.mp. | 1,319 |
| 23. | masitinib/ | 438 |
| 24. | Masitinib.mp. | 466 |
| 25. | lenvatinib/ | 1,355 |
| 26. | Lenvatinib.mp. | 1,400 |
| 27. | cabozantinib/ | 2,562 |
| 28. | Cabozantinib.mp. | 2,670 |
| 29. | sunitinib/ | 20,593 |
| 30. | Sunitinib.mp. | 21,125 |
| 31. | axitinib/ | 4,067 |
| 32. | Axitinib.mp. | 4,153 |
| 33. | pazopanib/ | 6,722 |
| 34. | Pazopanib.mp. | 6,879 |
| 35. | tivozanib/ | 474 |
| 36. | Tivozanib.mp. | 502 |
| 37. | vandetanib/ | 4,213 |
| 38. | Vandetanib.mp. | 4,341 |
| 39. | nintedanib/ | 2,126 |
| 40. | Nintedanib.mp. | 2,208 |
| 41. | regorafenib/ | 2,744 |
| 42. | Regorafenib.mp. | 2,847 |
| 43. | sorafenib/ | 25,721 |
| 44. | Sorafenib.mp. | 26,523 |
| 45. | dabrafenib/ | 3,268 |
| 46. | Dabrafenib.mp. | 3,433 |
| 47. | vemurafenib/ | 6,445 |
| 48. | Vemurafenib.mp. | 6,710 |
| 49. | encorafenib/ | 295 |
| 50. | Encorafenib.mp. | 304 |
| 51. | cobimetinib/ | 844 |
| 52. | Cobimetinib.mp. | 874 |
| 53. | trametinib/ | 3,718 |
| 54. | Trametinib.mp. | 3,810 |
| 55. | binimetinib/ | 552 |
| 56. | Binimetinib.mp. | 559 |
| 57. | everolimus/ | 24,301 |
| 58. | Everolimus.mp. | 26,840 |
| 59. | temsirolimus/ | 7,606 |
| 60. | Temsirolimus.mp. | 7,766 |
| 61. | abemaciclib/ | 568 |
| 62. | Abemaciclib.mp. | 580 |
| 63. | palbociclib/ | 2,151 |
| 64. | Palbociclib.mp. | 2,198 |
| 65. | ribociclib/ | 708 |
| 66. | Ribociclib.mp. | 737 |
| 67. | niraparib/ | 617 |
| 68. | Niraparib.mp. | 633 |
| 69. | olaparib/ | 3,493 |
| 70. | Olaparib.mp. | 3,603 |
| 71. | rucaparib/ | 801 |
| 72. | Rucaparib.mp. | 814 |
| 73. | vismodegib/ | 1,705 |
| 74. | Vismodegib.mp. | 1,775 |
| 75. | sonidegib/ | 589 |
| 76. | Sonidegib.mp. | 604 |
| 77. | cetuximab/ | 25,558 |
| 78. | Cetuximab.mp. | 26,316 |
| 79. | panitumumab/ | 7,398 |
| 80. | Panitumumab.mp. | 7,683 |
| 81. | pertuzumab/ | 3,931 |
| 82. | Pertuzumab.mp. | 4,083 |
| 83. | trastuzumab/ | 34,473 |
| 84. | Trastuzumab.mp. | 36,566 |
| 85. | trastuzumab emtansine/ | 2,113 |
| 86. | Trastuzumab emtansine.mp. | 2,189 |
| 87. | tamoxifen/ | 49,834 |
| 88. | Tamoxifen.mp. | 53,451 |
| 89. | fulvestrant/ | 7,848 |
| 90. | Fulvestrant.mp. | 8,037 |
| 91. | toremifene/ | 1,823 |
| 92. | Toremifene.mp. | 1,881 |
| 93. | megestrol acetate/ | 3,681 |
| 94. | Megestrol acetate.mp. | 3,811 |
| 95. | medroxyprogesterone acetate/ | 11,339 |
| 96. | Medroxyprogesterone acetate.mp. | 12,949 |
| 97. | anastrozole/ | 9,033 |
| 98. | Anastrozole.mp. | 9,210 |
| 99. | exemestane/ | 5,710 |
| 100. | Exemestane.mp. | 5,874 |
| 101. | letrozole/ | 10,862 |
| 102. | Letrozole.mp. | 11,108 |
| 103. | goserelin/ | 5,609 |
| 104. | Goserelin.mp. | 5,681 |
| 105. | buserelin/ | 2,424 |
| 106. | Buserelin.mp. | 3,087 |
| 107. | leuprorelin/ | 9,131 |
| 108. | Leuprorelin.mp. | 9,167 |
| 109. | Leuprolide.mp. | 2,203 |
| 110. | triptorelin/ | 4,254 |
| 111. | Triptorelin.mp. | 4,319 |
| 112. | anamorelin/ | 183 |
| 113. | Anamorelin.mp. | 194 |
| 114. | abiraterone/ | 3,327 |
| 115. | Abiraterone.mp. | 5,572 |
| 116. | abiraterone acetate/ | 2,330 |
| 117. | Abiraterone Acetate.mp. | 2,548 |
| 118. | bicalutamide/ | 5,589 |
| 119. | Bicalutamide.mp. | 5,728 |
| 120. | enzalutamide/ | 4,338 |
| 121. | Enzalutamide.mp. | 4,561 |
| 122. | flutamide/ | 6,280 |
| 123. | Flutamide.mp. | 6,535 |
| 124. | diethylstilbestrol/ | 5,394 |
| 125. | Diethylstilbestrol.mp. | 5,888 |
| 126. | interleukin.mp. | 564,845 |
| 127. | mifamurtide/ | 707 |
| 128. | Mifamurtide.mp. | 722 |
| 129. | talimogene laherparepvec/ | 626 |
| 130. | Talimogene laherparepvec.mp. | 651 |
| 131. | avelumab/ | 1,135 |
| 132. | Avelumab.mp. | 1,164 |
| 133. | nivolumab/ | 10,264 |
| 134. | Nivolumab.mp. | 10,680 |
| 135. | durvalumab/ | 1,896 |
| 136. | Durvalumab.mp. | 1,930 |
| 137. | ipilimumab/ | 10,466 |
| 138. | Ipilimumab.mp. | 10,820 |
| 139. | necitumumab/ | 355 |
| 140. | Necitumumab.mp. | 367 |
| 141. | pembrolizumab/ | 8,373 |
| 142. | Pembrolizumab.mp. | 8,723 |
| 143. | atezolizumab/ | 2,548 |
| 144. | Atezolizumab.mp. | 2,635 |
| 145. | dinutuximab/ | 230 |
| 146. | Dinutuximab.mp. | 238 |
| 147. | olaratumab/ | 249 |
| 148. | Olaratumab.mp. | 258 |
| 149. | bevacizumab/ | 51,869 |
| 150. | Bevacizumab.mp. | 53,282 |
| 151. | ramucirumab/ | 1,940 |
| 152. | Ramucirumab.mp. | 2,302 |
| 153. | aflibercept/ | 4,787 |
| 154. | Aflibercept.mp. | 4,928 |
| 155. | aldesleukin/ | 212 |
| 156. | Aldesleukin.mp. | 431 |
| 157. | degarelix/ | 687 |
| 158. | Degarelix.mp. | 723 |
| 159. | protein kinase inhibitor/ | 9,667 |
| 160. | Protein kinase inhibitor*.mp. | 25,072 |
| 161. | protein tyrosine kinase inhibitor/ | 28,485 |
| 162. | Protein tyrosine kinase inhibitor*.mp. | 28,885 |
| 163. | Tyrosine kinase inhibitor*.mp. | 50,599 |
| 164. | TKI.mp. | 15,510 |
| 165. | TKIs.mp. | 11,350 |
| 166. | Multireceptor tyrosine kinase inhibitor*.mp. | 13 |
| 167. | B Raf kinase inhibitor/ | 1,595 |
| 168. | B Raf kinase inhibitor*.mp. | 1,615 |
| 169. | MAPK inhibitor*.mp. | 4,758 |
| 170. | mitogen activated protein kinase inhibitor/ | 8,696 |
| 171. | Mitogen activated protein kinase inhibitor*.mp. | 9,120 |
| 172. | MEK inhibitor*.mp. | 7,524 |
| 173. | mammalian target of rapamycin inhibitor"/ | 10,191 |
| 174. | "Mammalian target of rapamycin inhibitor*".mp. | 10,944 |
| 175. | mTOR inhibitor*.mp. | 11,349 |
| 176. | CDK inhibitor*.mp. | 4,688 |
| 177. | cyclin dependent kinase inhibitor/ | 6,489 |
| 178. | Cyclin dependent kinase inhibitor*.mp. | 30,048 |
| 179. | nicotinamide adenine dinucleotide adenosine diphosphate ribosyltransferase inhibitor/ | 5,101 |
| 180. | nicotinamide adenine dinucleotide adenosine diphosphate ribosyltransferase inhibitor*.mp. | 5,103 |
| 181. | "Poly(ADP-ribose) polymerase inhibitor*".mp. | 837 |
| 182. | PARP inhibitor*.mp. | 5,062 |
| 183. | Hedgehog pathway inhibitor*.mp. | 442 |
| 184. | interleukin derivative/ | 4,093 |
| 185. | Interleukins.mp. | 7,979 |
| 186. | Anti-oestrogen*.mp. | 602 |
| 187. | antiestrogen*/ | 6,857 |
| 188. | antiestrogen.mp. | 8,332 |
| 189. | progesterone/ | 52,758 |
| 190. | Progesterone*.mp. | 93,935 |
| 191. | aromatase inhibitor/ | 12,922 |
| 192. | Aromatase inhibitor*.mp. | 16,109 |
| 193. | Gonadotropin-releasing hormone.mp. | 11,285 |
| 194. | Androgen antagonist*.mp. | 425 |
| 195. | antiandrogen/ | 9,750 |
| 196. | Antiandrogen*.mp. | 12,668 |
| 197. | androgen receptor antagonist/ | 804 |
| 198. | Androgen receptor antagonist*.mp. | 1,280 |
| 199. | estrogen/ | 77,835 |
| 200. | Estrogen*.mp. | 193,483 |
| 201. | Oestrogen*.mp. | 15,780 |
| 202. | Anti-gonadotrophin releasing hormone*.mp. | 4 |
| 203. | VEGF inhibitor*.mp. | 1,475 |
| 204. | Vascular endothelial growth factor inhibitor*.mp. | 424 |
| 205. | angiogenesis inhibitor/ | 16,797 |
| 206. | Angiogenesis inhibitor*.mp. | 18,898 |
| 207. | epidermal growth factor receptor kinase inhibitor/ | 7,515 |
| 208. | Epidermal growth factor receptor kinase inhibitor*.mp. | 7,555 |
| 209. | EGFR inhibitor*.mp. | 5,966 |
| 210. | anaplastic lymphoma kinase inhibitor/ | 722 |
| 211. | anaplastic lymphoma kinase inhibitor*.mp. | 815 |
| 212. | ALK inhibitor*.mp. | 1,986 |
| 213. | cMET inhibitor*.mp. | 77 |
| 214. | monoclonal antibody/ | 128,259 |
| 215. | monoclonal antibod*.mp. | 193,938 |
| 216. | MAB*.mp. | 52,680 |
| 217. | exp leukemia/ | 203,033 |
| 218. | Leukemia.mp. | 308,280 |
| 219. | Leukaemia.mp. | 30,062 |
| 220. | exp lymphoma/ | 209,181 |
| 221. | Lymphoma.mp. | 231,956 |
| 222. | exp rheumatoid arthritis/ | 143,804 |
| 223. | Rheumatoid arthritis.mp. | 156,410 |
| 224. | exp inflammatory bowel disease/ | 113,514 |
| 225. | Inflammatory bowel disease*.mp. | 72,223 |
| 226. | Ulcerative colitis.mp. | 60,175 |
| 227. | Crohn* disease.mp. | 77,747 |
| 228. | exp psoriasis/ | 63,401 |
| 229. | Psoriasis.mp. | 57,412 |
| 230. | exp ankylosing spondylitis/ | 19,351 |
| 231. | Ankylosing spondylitis.mp. | 22,848 |
| 232. | (Or/217 - 231) [OR for MAB uses that we are not interested in] | 837,019 |
| 233. | 214 or 215 or 216 [MAB synonyms] | 211,516 |
| 234. | 234. 233 not 232 [MABs but not disease states we are not interested in] | 178,024 |
| 235. | Targeted treatment*.mp. | 8,327 |
| 236. | Targeted technologies.mp. | 21 |
| 237. | Personalized treatment.mp. | 4,160 |
| 238. | Personalised treatment.mp. | 481 |
| 239. | Targeted therapy.mp. | 54,912 |
| 240. | Targeted therapies.mp. | 29,215 |
| 241. | Precision medicine.mp. | 8,630 |
| 242. | Molecularly targeted drug*.mp. | 395 |
| 243. | molecularly targeted therapy/ | 27,560 |
| 244. | Molecular targeted therap*.mp. | 3,761 |
| 245. | personalized medicine/ | 33,557 |
| 246. | Personalized medicine.mp. | 39,734 |
| 247. | Personalised medicine.mp. | 1,727 |
| 248. | Tailored medicine.mp. | 104 |
| 249. | Genomic medicine.mp. | 1,445 |
| 250. | Precision cancer care.mp. | 31 |
| 251. | Stratified medicine.mp. | 437 |
| 252. | (Or/1 – 213) or (Or/234 – 251) [Targeted Therapy OR] | 1,412,394 |
| 253. | (Molecular adj2 test).mp. | 2,508 |
| 254. | Genomic testing.mp. | 966 |
| 255. | Targeted test*.mp. | 501 |
| 256. | biomarker test*.mp. | 1,401 |
| 257. | molecular test*.mp. | 9,599 |
| 258. | mutation* test*.mp. | 4,099 |
| 259. | Test* trend*.mp. | 268 |
| 260. | Genetic profile.mp. | 3,145 |
| 261. | Genetic testing.mp. | 28,563 |
| 262. | pharmacogenetic testing/ | 623 |
| 263. | Pharmacogenetic testing.mp. | 1,319 |
| 264. | Pharmacogenomic testing.mp. | 353 |
| 265. | Molecular profil*.mp. | 10,439 |
| 266. | Molecular diagnostics.mp. | 7,419 |
| 267. | Genotyping.mp. | 79,715 |
| 268. | Genomic profiling.mp. | 3,262 |
| 269. | Gene test*.mp. | 2,238 |
| 270. | Molecular diagnostic techniques.mp. | 598 |
| 271. | (EGFR adj2 test*).mp. | 1,972 |
| 272. | HER2 test*.mp. | 962 |
| 273. | ALK test*.mp. | 274 |
| 274. | BRAF test*.mp. | 240 |
| 275. | ER test*.mp. | 81 |
| 276. | PR test*.mp. | 140 |
| 277. | KIT test*.mp. | 168 |
| 278. | BRCA test*.mp. | 621 |
| 279. | RET test*.mp. | 34 |
| 280. | MET test*.mp. | 55 |
| 281. | KRAS test*.mp. | 363 |
| 282. | NRAS test*.mp. | 18 |
| 283. | PD1 test*.mp. | 1 |
| 284. | CTLA4 test*.mp. | 1 |
| 285. | (Or/253 – 284) [Molecular Testing OR] | 150,822 |
| 286. | Receipt of.mp. | 22,756 |
| 287. | Treatment with.mp. | 554,115 |
| 288. | Receive*.mp. | 1,315,723 |
| 289. | Non-use.mp. | 3,195 |
| 290. | Treatment receipt.mp. | 200 |
| 291. | Receipt.mp. | 22,766 |
| 292. | Initiat*.mp. | 704,192 |
| 293. | Treatment utilisation.mp. | 77 |
| 294. | Utili#ation.mp. | 290,352 |
| 295. | Access.mp. | 400,446 |
| 296. | Underutilisation.mp. | 254 |
| 297. | Underutilization.mp. | 2,326 |
| 298. | Treatment barrier*.mp. | 628 |
| 299. | Non receipt.mp. | 152 |
| 300. | Non-initiation.mp. | 113 |
| 301. | Treatment pattern*.mp. | 7,916 |
| 302. | Utilization pattern*.mp. | 3,461 |
| 303. | Utilisation pattern*.mp. | 325 |
| 304. | drug utilization/ | 16,809 |
| 305. | Drug utilization.mp. | 18,761 |
| 306. | Drug utilisation.mp. | 884 |
| 307. | Uptake.mp. | 360,750 |
| 308. | Provision.mp. | 78,138 |
| 309. | (Or/286 – 308) [Utilization OR] | 3,314,589 |
| 310. | Socioeconomic status.mp. | 39,767 |
| 311. | Socioeconomic factor*.mp. | 9,588 |
| 312. | poverty/ | 35,806 |
| 313. | Poverty.mp. | 44,341 |
| 314. | Poverty areas.mp. | 196 |
| 315. | social class/ | 21,008 |
| 316. | Social class.mp. | 23,178 |
| 317. | Social mobility.mp. | 511 |
| 318. | Index of multiple deprivation.mp. | 911 |
| 319. | Socioeconomic position.mp. | 2,926 |
| 320. | Carstairs index.mp. | 60 |
| 321. | Townsend index.mp. | 101 |
| 322. | Area level deprivation.mp. | 170 |
| 323. | Poverty level.mp. | 1,859 |
| 324. | socioeconomics/ | 101,475 |
| 325. | Socioeconomic.mp. | 86,230 |
| 326. | Socioeconomics.mp. | 101,733 |
| 327. | Income level.mp. | 2,729 |
| 328. | (Income adj6 category).mp. | 419 |
| 329. | household income/ | 3,729 |
| 330. | Household income.mp. | 11,616 |
| 331. | Median adj2 income).mp. | 2,792 |
| 332. | Education status.mp. | 976 |
| 333. | (Education adj6 demographic).mp. | 2,862 |
| 334. | (Education adj6 variable).mp. | 1,062 |
| 335. | Education level.mp. | 17,048 |
| 336. | Employment status.mp. | 15,256 |
| 337. | Employment characteristic*.mp. | 206 |
| 338. | Employment adj6 variable).mp. | 182 |
| 339. | Unemployed.mp. | 9,462 |
| 340. | Unemploy* adj6 variable).mp. | 48 |
| 341. | Disab* adj6 variable).mp. | 906 |
| 342. | Race ethnicity.mp. | 24,035 |
| 343. | (Race adj6 variable).mp. | 672 |
| 344. | Ethnicity adj6 variable).mp. | 435 |
| 345. | Race.ti,ab. | 125,659 |
| 346. | Racial.ti,ab. | 40,988 |
| 347. | Smoker*.mp. | 104,222 |
| 348. | Non-smoker*.mp. | 19,714 |
| 349. | (Smoking adj2 history).mp. | 21,857 |
| 350. | Health insurance status.mp. | 1,049 |
| 351. | exp medicare/ | 60,158 |
| 352. | Medicare.mp. | 70,152 |
| 353. | medicaid/ | 34,820 |
| 354. | Medicaid.mp. | 41,596 |
| 355. | Insurance coverage.mp. | 8,902 |
| 356. | Health plan type.mp. | 111 |
| 357. | Public insurance.mp. | 1,805 |
| 358. | medically uninsured/ | 4,169 |
| 359. | Medically uninsured.mp. | 4,286 |
| 360. | Insurance adj6 variable).mp. | 263 |
| 361. | Commercial insurance.mp. | 1,357 |
| 362. | private health insurance/ | 4,141 |
| 363. | Private health insurance.mp. | 5,222 |
| 364. | Military insurance.mp. | 35 |
| 365. | Other insurance.mp. | 314 |
| 366. | (Or/310 – 365) [Socio-economic Status] | 580,384 |
| 367. | 252 or 285 [Targeted therapy or molecular testing] | 1,541,395 |
| 368. | 367 and 309 and 366 Targeted therapy or molecular testing – utilization and  Socio-economic status] | 7,203 |
| 369. | limit 368 to yr=“1998 -current” | 7,137 |

**C. Scopus search strategy 05/02/2019 (kept updated through to 31/12/2019)**

|  | **Search term** | **Number Retrieved** |
| --- | --- | --- |
| 1. | Alectinib” OR “Ceritinib” OR “Crizotinib” OR “Brigatinib” OR “Erlotinib” OR “Gefitinib” OR “Afatinib” OR “Osimertinib” OR “Imatinib” OR “Lapatinib” OR “Neratinib” OR “Masitinib” OR “Lenvatinib” OR “Cabozantinib” OR “Sunitinib” OR “Axitinib” | 78,221 |
| 2. | “Pazopanib” OR “Tivozanib” OR “Vandetanib” or “Nintedanib” OR “Regorafenib” OR “Sorafenib” OR “Dabrafenib” OR “Vemurafenib” OR “Encorafenib” OR “Cobimetinib” OR “Trametinib” OR “Binimetinib” OR “Everolimus” OR “Temsirolimus” OR “Abemaciclib” | 47,541 |
| 3. | Palbociclib” OR “Ribociclib” OR “Niraparib” OR “Olaparib” OR “Rucaparib” OR “Vismodegib” OR “Sonidegib” OR “Cetuximab” OR “Pantimumab” OR “Pertuzumab” OR “Trastuzumab” OR “Trastuzumab Emtansine” OR “Tamoxifen” OR “Fulvestrant” OR “Toremifene” | 106,598 |
| 4. | “Megestrol Acetate” OR “Medroxyprogesterone Acetate” OR “Anastrozole” OR “Exemestane” OR “Letrozole” OR “Goserelin” OR “Buserelin” OR “Leuprorelin Acetate” OR “Leuprolide” OR “Triptorelin Pamoate” OR “Triptorelin” OR “Anamorelin” OR “Abiraterone Acetate” | 52,197 |
| 5. | “Bicalutamide” OR “Enzalutamide” OR “Flutamide” OR “Diethylstilbestrol” OR “Interleukin” OR “Mifamurtide” OR “Talimogene Laherparepvec” OR “Avelumab” OR “Nivolumab” OR “Durvalumab” OR “Ipilimumab” OR “Necitumumab” OR “Pembrolizumab” OR “Atezolizumab” | 565,325 |
| 6. | “Dinutuximab” OR “Olaratumab” OR “Bevacizumab” OR “Ramucirumab” OR “Aflibercept” OR “Aldesleukin” OR “Degarelix” | 45,136 |
| 7. | “Protein Kinase Inhibitor*” OR “Tyrosine Kinase Inhibitor*” OR “Tyrosine Protein Kinase Inhibitor*” OR “TKI” or “TKIs” OR “Multireceptor Tyrosine Kinase Inhibitor*” OR “BRAF Kinase Inhibitor*” OR “MAPK Inhibitor” | 86,043 |
| 8. | “Mitogen-activated Protein Kinase Inhibitor*” OR “mTOR Inhibitor*” OR “CDK Inhibitor*” OR “Cyclin Dependent Kinase Inhibitor*” OR “Poly(ADP-ribose) Polymerase Inhibitor*” OR “PARP Inhibitor*” OR “Hedgehog Pathway Inhibitor*” OR “Interleukins” | 76,259 |
| 9. | “Anti-oestrogen*” OR “Progesterone” OR “Aromatase Inhibitor*” OR “Gonadotrophin-Releasing Hormone*” OR “Androgen Antagonist*” OR  “Anti-androgen*” OR “Androgen Receptor Antagonist*” OR “Estrogen*” OR “Oestrogen*” OR “Anti-gonadotrophin Releasing Hormone*” | 375,362 |
| 10 | “VEGF Inhibitor*” OR “Vascular Endothelial Growth Factor Inhibitor*” OR “Angiogenesis Inhibitor*” OR “EGFR Inhibitor*” OR “Epidermal Growth Factor Inhibitor*” OR “ALK Inhibitor*” OR “cMET Inhibitor*” | 36,188 |
| 11. | “Monoclonal Antibod*” OR “MAB” | 307,997 |
| 12. | “Leukemia” OR “Leukaemia” OR “Lymphoma” OR “Rheumatoid Arthritis” OR “Inflammatory Bowel Diseases” OR “Ulcerative Colitis” OR “Crohn* Disease” OR “Psoriasis” OR “Ankylosing Spondylitis” | 982,757 |
| 13. | (“Monoclonal Antibod*” OR “MAB”) AND NOT (#12) [MABs but not disease states that we are not interested in] | 265,890 |
| 14. | “Targeted Treatment*” OR “Targeted Technologies” OR “Personalised Treatment” OR “Personalized Treatment” OR “Targeted Therapy” OR “Targeted Therapies” OR “Precision Medicine” OR “Molecularly Targeted Drug*” OR “Molecularly Targeted Therap*” | 87,157 |
| 15. | “Personalised Medicine” OR “Personalized Medicine” OR “Tailored Medicine” OR “Genomic Medicine” OR “Precision Cancer Care” OR “Stratified Medicine” | 37,306 |
| 16. | (#1) OR (#2) OR (#3) OR (#4) OR (#5) OR (#6) OR (#7) OR (#8) OR (#9) OR (#10) OR (#13) OR (#14) OR (#15) [Targeted Therapies OR] | 1,496,121 |
| 17. | “Genomic Testing” OR “Targeted Test*” OR “Biomarker Test*” OR “Molecular Test*” OR “Mutation* Test*” OR “Test* Trend*” OR “Test* Pattern” OR “Genetic Profile” OR “Genetic Testing” OR “Pharmacogenomic Testing*” OR “Pharmacogenetic Testing” | 58,536 |
| 18. | “Molecular Profil*” OR “Molecular Diagnostics” OR “Genotyping” OR “Genomic Profiling” OR “Gene Test*” OR “Molecular Diagnostic Techniques” OR “EGFR Test*” OR “HER2 Test*” OR “ALK Test*” OR “BRAF Test*” OR “ER Test*” OR “PR Test*” OR “KIT Test*” | 99,486 |
| 19. | “BRCA Test*” OR “RET Test*” OR “MET Test*” OR “KRAS Test*” OR “NRAS Test*” OR “PD1 Test*” OR “CTLA4 Test*” | 597 |
| 20. | (#17) OR (#18) OR (#19) [Molecular Testing OR] | 153,771 |
| 21. | “Receipt of” OR “Treatment with” OR “Receive*” OR “Non-use” OR “Treatment Receipt” OR “Receipt” OR “Initiat*” OR “Treatment Utilisation” OR “Treatment Utilization” OR “Utilization” OR “Utilisation” OR “Access” OR “Underutilisation” OR “Underutilization” | 5,132,936 |
| 22. | “Treatment Barrier*” OR “Non-receipt” OR “Non-initiation” OR “Treatment Pattern*” OR “Utilisation Pattern*” OR “Utilization Pattern*” OR “Drug Utilization” OR “Drug Utilisation” OR “Uptake” OR “Provision” | 848,630 |
| 23. | (#21) OR (#22) [Utilization synonyms] | 5,816,262 |
| 24. | “Socioeconomic Status” OR “Socioeconomic Factors” OR “Poverty” OR “Poverty Areas” OR “Social Class” OR “Social Mobility” OR “Index of Multiple Deprivation” OR “Socioeconomic Position” OR “Carstairs Index” OR “Townsend Index” OR “Area Level Deprivation” | 335,186 |
| 25. | “Poverty Level” OR “Income Level” Or “Household Income” “Education Status” OR “Education Level” OR “Employment Status” OR “Employment Characteristic*” OR “Unemployed” OR “Race Ethnicity” OR “Smoker*” OR “Non-smoker*” OR “Smoking History” | 3,630 |
| 26. | “Health Insurance Status” OR “Socioeconomic*” OR “Medicare” OR “Medicaid” OR “Insurance Coverage” OR “Health Plan Type” OR “Public Insurance” OR “Medically Uninsured” OR “Commercial Insurance” OR “Private Insurance” OR “Military Insurance” | 415,616 |
| 27. | “Other Insurance” OR “Income Category” OR “Median Income” OR “Education Demographic” OR “Education Variable” OR “Employment Variable” OR “Unemploy* Variable” OR “Disab* Variable” OR “Race Variable” OR “Ethnicity Variable” OR  “Insurance Variable” | 2,670 |
| 28. | (#24) OR (#25) OR (#26) OR (#27) [Socio-economic status OR] | 554,623 |
| 29. | (#16) OR (#20) [Targeted therapy or molecular testing OR] | 1,633,613 |
| 30. | (#29) AND (#23) AND (#28) [Targeted therapy or molecular testing – and utilization socio-economic status]  Search filtered for date: 1998 onwards. | 1,916 |

**Supplementary Methods 3: Full text study inclusion/exclusion criteria decision trees**

**A. Predictive biomarker test articles**

**B. Biological and precision therapy articles**

**Supplementary Methods 4: Quality Appraisal Tool**

| **Question** | **Appraisal** | **Yes = 1** | **No = 0** | **Unclear = 0** |
| --- | --- | --- | --- | --- |
|  | ***Data Sources*** | | | |
| 1 | Did the author(s) address issues regarding completeness of SES and treatment data or consistency of coding SES and treatment data in the registry/database?  *Missing data is addressed.*  *Details of data source given.*  *2 points = Tick*  *1 point = Half tick* |  |  |  |
|  | ***Methods - Study Population & Variables*** | | | |
| 2 | Was the study subjects and the setting described in detail?  *Explicit statement for inclusion/exclusion criteria given e.g. cancer type, time period, staging, location, age.*  *Total study population number of interest stated (explicit statement, on consort diagram or total listed in table - not requiring calculation).*  *2 points = Tick*  *1 point = Half tick* |  |  |  |
|  | ***Methods - Operational Definitions*** | | | |
| 3 | Was the exposure (SES) measure clear?  *Unit of measure is stated.*  *Clear which SES rank is high or low.*  *2 points = Tick*  *1 point = Half tick* |  |  |  |
| 4 | Were the outcome measure (drug or test utilization) clear?  *Comparator(s) reported e.g. no precision medicine/biological and/or predictive biomarker test or a clinical alternative e.g. chemotherapy.*  *Drug code identification in registry listed or enough detail provided to identify where this information came from.*  *2 points = Tick*  *1 point = Half tick* |  |  |  |
|  | ***Results & Statistics*** | | | |
| 5 | Is utilization data of interest reported in tables as patient numbers (not just percent)?  *Yes = Tick*  *No = No tick* |  |  |  |
| 6 | Were the SES and treatment groups that were statistically compared related to data of interest?  *P value compares SES differences between the treatment groups of interest (e.g. no precision medicine/biological and/or predictive biomarker test or a clinical alternative e.g. chemotherapy).*  *Describes the statistical test used to compare SES difference in treatment groups (e.g. Chi square).*  *2 points = Tick*  *1 point = Half tick* |  |  |  |
| 7 | Has the association between exposure and outcome being statistically analysed (e.g. OR, RR) and this reported for the variables of interest?  Yes = Tick  No = No tick |  |  |  |
| 8 | Control methods: Did the authors use a method to control for confounders within the data of interest?  *Adjusted analysis is carried out for the data of interest e.g. multivariate analysis, PSM.*  *Confounders are listed.*  *2 points = Tick*  *1 point = Half tick* |  |  |  |
|  | ***Discussion/Conclusions*** | | | |
| 9 | Have the author(s) discussed SES and treatment utilization findings?  *Yes = Tick*  *No = No tick* |  |  |  |
| 10 | Have the authors acknowledged limitations that may reduce the generalizability of the results to other populations and settings?  *Yes = Tick (Clear - both limitations and generalizability addressed).*  *Yes= Half a tick (Ambiguous, more reliant on ‘assumptions’ than explicit statements).*  *No = No tick* |  |  |  |
|  | Overall Score |  | | |

**Table S1: Characteristics of studies reporting predictive biomarker test and/or biological and precision therapies utilization without a denominator population or which only reported a measure of average socio-economic status.**

|  | **Sampling Frame** | | | | | **SES** | | **Utilization by SES Grouping (Number, %)** | | | | | | |
| --- | --- | --- | --- | --- | --- | --- | --- | --- | --- | --- | --- | --- | --- | --- |
| **Study** | **Country** | **Data Source** | **Study Population^a^** | **Predictive Biomarker Test/**  **Biological and Precision Medicine**  **Overall Utilization (Number, %)** | **Comparator** | **Unit** | **Measure** | **Lowest SES Group** | | **Highest SES Group** | | | | **QA** |
| **Breast Cancer: Biological & Precision Therapies** | | | | | | | | | | | | | | |
| Freedman *et al.* (2014) | USA | SEER-Medicare | 28% of US Population  2005 - 2009  Age ≥ 66  Stage I - III  n = 2,106 | Concurrent or Sequential Trastuzumab (with Either Standard or Non-Standard Chemotherapy)  n = 2,106 (100.0) | None | Census Tract | Median Household Income | Q1 (Low)  514/2,106  (24.4) | Q2  534/2,106  (25.4) | Q3  528/2,106  (25.1) | Q4 (High)  530/2,106  (25.2) |  | | 5 |
|  |  |  |  |  |  | Census Tract | % With High School Diplomas | Q1 (Low)  527/2,106  (25.0) | Q2    529/2,106  (25.1) | Q3  524/2,106  (24.9) | Q4 (High)  526/2,106  (25.0) |  | |  |
| Vaz- Luis *et al.* (2014) | USA | SEER-Medicare | 28% of US Population  06/2005 - 12/2009  Age ≥ 66  Stage I - III  n = 2,028 | Trastuzumab  n = 2,028 (100.0) | None | Census Tract | Median Household Income | Q1 (Low)  486/2,028  (24.0) | Q2  522/2,028  (25.7) | Q3  508/2,028  (25.1) | Q4 (High)  512/2,028  (25.3) |  | | 5 |
|  |  |  |  |  |  | Census Tract | % With a High School Diploma | Q1 (Low)  503/2,028  (24.8) | Q2:  508/2,028  (25.1) | Q3  508/2,028  (25.1) | Q4 (High)  509/2,028  (25.1) |  | |  |
| Reeder - Hayes *et al.* (2017) | USA | SEER-Medicare | 25% of US Population  2005 - 2011  Age ≥ 66  Stage I - III  n = 1,077  n (PSM) = 416 | Trastuzumab  (With Doxorubicin, Cyclophosphamide & Paclitaxel or Docetaxel & Carboplatin Regimens)  n = 1,077 (100.0)  n (PSM) = 416 (100.0) | None | Census Tract | Residents Living Below the Poverty Line (%) | ≥ 20% (Low)  141/1,077  (13.1) | 10% - 19.99%  288/1,077  (26.7) | 5% -9.99%  288/1,077  (26.7) | 0% - 4.99%  (High)  360/1,077  (33.4) | | | 5 |
|  |  |  |  |  |  |  |  | PSM  53/416 (12.7) | PSM  106/416  (25.5) | PSM  117/416  (28.1) | PSM  140/416  (33.6) | | |  |
|  | **Sampling Frame** | | | | | **SES** | | **Utilization by SES Grouping (Number, %)** | | | | | | |
| **Study** | **Country** | **Data Source** | **Study Population^a^** | **Predictive Biomarker Test/ Biological and Precision Medicine**  **Overall Utilization (Number, %)** | **Comparator** | **Unit** | **Measure** | **Lowest SES Group Highest SES Group** | | | | | | **QA** |
| Chavez- MacGregor *et al.* (2015) | USA | SEER-Medicare & Texas Cancer Registry-Medicare Linked Databases | 28% of US Population and Texas State  2005 - 2009  Age ≥ 66  Stage I - III  n = 2,203 | Trastuzumab  n = 2,203 (100.0) | None | NR  NR | Education | 4^th^ (Low)  561/2,203  (25.5) | 3^rd^  545/2,203  (24.7) | 2^nd^  547/2,203  (24.8%) | 1^st^ (High)  550/2,203  (25.0) |  | | 4.5 |
|  |  |  |  |  |  |  | Poverty | 1^st^ (Low)  550/2,203  (25.0) | 2^nd^  546/2,203  (24.8) | 3^rd^  546/2,203  (24.8) | 4^th^ (High)  561/2,203  (25.5) |  | |  |
| Lu *et al.* (2013) | Australia | Medicare  Australia (Administering Body of the Nationally Funded Herceptin Program, PBS and MBS) | Women Enrolled in the Herceptin Program  12/2001 – 03/2010  Stage: Metastatic HE2+ Cancer  N = 3,418 | Trastuzumab  n = 3,418 (100.0) | None | Census | Australia’s IRSD (Includes Income, Education Attainment & Unemployment Information) | Q1 (Low)  426/3,418  (12.5) | Q2  787/3,418  (23.0) | Q3  644/3,418  (18.8) | Q4 Q5 (High)  789/3,418 772/3,418  (23.1) (22.6) | | | 3.5 |
| **Melanoma: Biological & Precision Therapies** | | | | | | | | | | | | | | |
| Krimphove *et al.* (2019) | USA | NCDB | 1,500 CoC Accredited High & Low Immunotherapy Prescribing Hospitals  2011 - 2015  Age ≥ 20  Stage: IV  n = 1,863 | Immunotherapy  n = 1,863 (100.0) | None | Zip Code | Median Household Income | < $37,000 (Low)  215/1,863 (11.5) | $38,000 - $47,999  412/1,863  (22.1) | $48,000 - $62,999  516/1,863  (27.7) | ≥ $63,000+ (High)  714/1,863 (38.3) | |  | 6.5 |
|  |  |  |  |  |  | Zip Code | % of Adults Without a High School Diploma | ≥ 21% (Low)  258/1,863 (13.8) | 13% - 20%  422/1,863  (22.7) | 7% - 12%  637/1,863 (34.2) | < 7% (High)  542/1,863  (29.1) | | |  |
|  | **Sampling Frame** | | | | | **SES** | | **Utilization by SES Grouping (Number, %)** | | | | | | |
| **Study** | **Country** | **Data Source** | **Study Population^a^** | **Predictive Biomarker Test/ Biological and Precision Medicine**  **Overall Utilization**  **(Number, %)** | **Comparator** | **Unit** | **Measure** | **Lowest SES Group** | | **Highest SES Group** | | | | **QA** |
| **Colon Cancer: Biological & Precision Therapies** | | | | | | | | | | | | | | |
| Zheng *et al.* (2014) | USA | SEER - Medicare | 2003 - 2007  Age ≥ 66  Stage: Metastatic  n = 7,895 | Chemotherapy & Biologics  n = 1,260 (16.0) | No Treatment/  Fluorouracil, Capecitabine, Floxuridine, Leucovorin & levoleucovorin (5FU/LV)/ Oxaliplatin, Irinotecan or Both | Zip Code | Household Median Income | Biologics  Mean: $51,000 SD $23,000 | No Treatment  Mean:  $48,000  SD $23,000 | 5FU/LV Oxaliplatin  Irinotecan  or Both  Mean: Mean:  $51,000 $52,000  SD $24,000 SD $25,000 | | | | 5 |
| **Hepatocellular Cancer: Biological & Precision Therapies** | | | | | | | | | | | | | | |
| Kwan *et al.* (2018) | USA | SEER - Medicare | 26% of the US Population  01/2007 - 12/2011  Age ≥ 65  Stage I - IV  n = 1,017 | Sorafenib  n = 369 (36.3) | Embolization | Census Tract | Median Income | Sorafenib  Median:  $26,607  SD $22,558 | Embolization  Median:  $51,774  SD $25,273 |  |  |  | | 7 |
|  |  |  |  |  |  |  |  | P Value: **< 0.001** | | | | | |  |
|  |  |  |  |  |  |  |  | PSM:  Median:  $49,445 | PSM:  Median:  $48,761 |  |  |  | |  |
|  |  |  |  |  |  |  |  | Standardized Difference = 3% | | | | | |  |
| **Renal Cell Carcinoma: Biological & Precision Therapies** | | | | | | | | | | | | | | |
| Li *et al.* (2019) | USA | SEER-Medicare | 20 US Registries  (28% of US Population)  2000 - 2013  Stage IV  n = 1,015 | Targeted Therapy  (Sorafenib, Sunitinib, Temsirolimus, Everolimus, Bevacizumab, Pazopanib & Axitinib)  n = 641 (63.2) | Non-Targeted Therapy | County level | Per Capita Income | Targeted Therapy  Mean: $39,900  SD: $10,700 | Non Targeted Therapy  Mean: $31,400  SD: $9,900 |  |  |  | | 5 |
|  |  |  |  |  |  |  |  | P Value: < **0.001** | | | | | |  |
|  |  |  |  |  |  | County level | Unemployment Rate Per 1,000 Residents | Targeted Therapy  Mean: 39.4  SD 16.1 | Non Targeted Therapy  Mean: 27.0  SD 10.5 |  |  |  | |  |
|  |  |  |  |  |  |  |  | P Value: < **0.001** | | | | | |  |
|  | **Sampling Frame** | | | | | **SES** | | **Utilization by SES Group (Number, %)** | | | | | |  |
| **Study** | **Country** | **Data Source** | **Study Population^a^** | **Predictive Biomarker Test/ Biological and Precision Medicine**  **Overall Utilization (Number, %)** | **Comparator** | **Unit** | **Measure** | **Lowest SES Group** | | **Highest SES Group** | | | | **QA** |
| **Head & Neck Cancer: Biological & Precision Therapies** | | | | | | | | | | | | | | |
| Zandberg *et al.* (2018) | USA | SEER - Medicare | 17 Registries (28% of US Population)  2005 - 2011  Squamous Cell  Age ≥ 66  n = 2,135 | Cetuximab with Concurrent Radiation  n = 579 (27.1) | Radiation with Concurrent Cytotoxic Chemotherapy/Radiation | Census Tract | Median Income | Cetuximab with Concurrent Radiation  Median: $51,000  Range: $8,000 - $232,000 | Radiation with Cytotoxic Chemotherapy  Median: $49,000  Range:  $9,000 - $250,000 | Radiation  Median: $46,000  Range:  $11,000 - $250,000 |  |  | | 5.5 |

^a^Refers to the total number of patients in the cohort of interest.

Utilization number (%) reported.

**P Values** = Significant at P < 0.05

Quality appraisal scores range from 0 (lowest) to 10 (highest).

Abbreviations: CoC: Commission on Cancer; IRSD: Index of Relative Socio-economic Disadvantage; MBS: Medicare Benefits Scheme; NCDB: National Cancer Database; NR: Not reported; PBS: Pharmaceutical Benefits Scheme; PSM: Propensity Score Matching; QA: Quality Appraisal; SEER: Surveillance, Epidemiology and End Results Program; SD: Standard Deviation; SES: socio-economic status.

**Figure S1: Forest plot of odds of biological and precision therapy utilization in SEER versus Non-SEER registry studies for low compared to high socio-economic status (including funnel plot)**

Figure S1: Funnel plot of biological and precision therapy utilization for SEER versus non-SEER studies.

**Table S2: Characteristics of included studies**

|  | **Sampling Frame** | | | | | **SES** | | **Utilization by SES Grouping (Number, %)** | | | | | | |
| --- | --- | --- | --- | --- | --- | --- | --- | --- | --- | --- | --- | --- | --- | --- |
| **Study** | **Country** | **Data Source** | **Study Population^a^** | **Predictive Biomarker Test /Biological and Precision Therapies Overall Utilization (Number, %)** | **Comparator** | **Unit** | **Measure** | **Lowest SES Group** | | **Highest SES Group** | | | **Utilization in Low SES (OR & 95% CI)^b^** | **QA** |
| **Predictive Biomarker Testing** | | | | | | | | | | | | | | |
| Pensa *et al.* (2009)* | USA | Connecticut Tumor Registry - SEER Database | Connecticut  2000 - 2003  Breast Cancer  n = 1,364 | HER2 Test  n = 894 (65.5) | No HER2 Test | Census | Median Household Income of Town | Lowest Tertile (Low)  299/462 (64.7) | Middle  /Highest Tertile (High)  595/902 (66.0) |  |  |  | 0.95  (0.74 - 1.21) | 5.5 |
|  |  |  |  |  |  |  |  | OR: 0.94  (0.75 - 1.20) | OR: Ref |  |  |  |  |  |
| Lund *et al.* (2010)* | USA | Atlanta SEER Registry & Georgia Comprehensive Cancer Registry | Fulton and Dekalb Counties in Metropolitan Atlanta  2003 - 2004  Stage I - IV  Breast Cancer  n = 1,842 | HER2 Test  n = 1,660 (90.1) | No HER2 Assay | Census Tract | % Living Below the Federally Defined Poverty Line | ≥ 20% (Low)  286/319 (89.7) | 10 < 20%  390/433 (90.1) | 5 < 10%  435/479 (90.8) | < 5% (High)  548/607 (90.3) |  | 0.93  (0.58 - 1.51) | 6.5 |
|  |  |  |  |  |  |  |  | P Value: 0.9565 | | | | |  |  |
| Ferrusi *et al.* (2013)* | Canada | OCR | Ontario  01/2006 - 12/2007  Early Stage  (I - III)  Breast Cancer  n = 13,396 | Documented HER2 Test  n = 8,854 (66.1) | Undocumented HER2 Test | Census Tract (Postcode) | Household Income | Q1 (Low)  1,563/2,334 (67.0) | Q2  1,667/2,577 (64.7) | Q3  1,770/2,649 (66.8) | Q4  1,856/2,812 (66.0) | Q5 (High)  1,977/2,991 (66.1) | 1.04  (0.93 - 1.17) | 8.5 |
|  |  |  |  |  |  |  |  | Q1, 2, 3 or 4 vs Q5  OR: 1.00 (0.85 - 1.17) | | | | |  |  |
|  |  |  |  |  |  |  |  | P Value: Not significant at 0.05 | | | | |  |  |
| de Camargo Cancela *et al.* (2015)* | Ireland | National Cancer Registry Ireland | Ireland  2006 - 2008  Stage I - IV  Breast Cancer  n = 7,619^c^ | HER2 Test  n = 6,529 (85.7) | ER Test/  PR Test/  Any Hormone Receptor Tests | Area of Residence | Deprivation Status | Q1 (Low)  1,697/1,990 (85.3)^c^ | Q2  970/1,135 (85.5)^c^ | Q3  827/986 (83.9)^c^ | Q4  904/1,042 (86.8)^c^ | Q5 (High)  1,476/1,691 (87.3)^c^ | 0.84  (0.69 - 1.02) | 7 |
|  |  |  |  |  |  |  |  | IRR (No HER2 Test): 1.15  (0.98 - 1.36) | IRR (No HER2 Test): 1.14 (0.95 - 1.38) | IRR (No HER2 Test): 1.27  (1.05 - 1.53) | IRR (No HER2 Test): 1.04 (0.85 - 1.27) | IRR (No HER2 Test): Ref |  |  |
|  | **Sampling Frame** | | | | | **SES** |  | **Utilization by SES Grouping (Number, %)** | | | | |  |  |
| **Study** | **Country** | **Data Source** | **Study Population^a^** | **Predictive Biomarker Test/ Biological and Precision Therapies Overall Utilization (Number, %)** | **Comparator** | **Unit** | **Measure** | **Lowest SES Group** | | **Highest SES Group** | | | **Utilization in Low SES (OR & 95% CI) ^b^** | **QA** |
| Greenbaum *et al.* (2017)* | USA | New Mexico Tumour Registry  Participating in SEER | New Mexico Residents  2010 - 2013  Stage IV  Colorectal Cancer  n = 637 | KRAS Test  n = 245  (38.5) | NR | Census Tract | Annual Income per capita | $5,051 - $15,656 (Low)  52/153 (34.0) | $15,662 - $23,034  62/153 (40.5) | $23,126 - $32,042  60/153 (39.2) | $32,138 –  $84,620 (High)  61/152 (40.1) | | 0.77  (0.47 - 1.26) | 8.5 |
|  |  |  |  |  |  |  |  | OR: Ref | OR: 1.48 (0.88 - 2.48) | OR: 1.27 (0.68 - 2.35) | OR: 1.21 (0.63 - 2.34) |  |  |  |
|  |  |  |  |  |  |  |  | P Value: 0.618 | | | | |  |  |
| Rico *et al.* (2016)* | USA | 10 Centers for Disease Control & Prevention National Program of Central Cancer Registries | Alaska, California (13 Counties in Sacramento area), Colorado, Florida (5 Miami Metro Counties), Idaho, Louisiana, New Hampshire, North Carolina, Rhode Island & Texas.  2011  Stage: Metastatic  Colorectal Cancer  n = 3,608 | KRAS Test  n = 992 (27.5) | Not Tested | Census Tract | % People Living Under the Federal Poverty Level | ≥ 20% (Low)  251/968  (25.9) | < 20% (High)  733/2,619 (28.0) |  |  |  | 0.90  (0.76 - 1.07) | 9.5 |
|  |  |  |  |  |  |  |  | P Value: 0.2201 | | | | |  |  |
|  |  |  |  |  |  |  |  | OR: 1.13  (0.91 - 1.40) | OR: Ref |  |  |  |  |  |
|  |  |  |  |  |  |  |  | P Value: 0.2665 | | | | |  |  |
|  |  |  |  |  |  | Census Tract | % Without High School Education | ≥ 25% (Low)  212/902 (23.5) | < 20% (High)  773/2,689 (28.7) | |  |  | 0.76  (0.64 - 0.91) |  |
|  |  |  |  |  |  |  |  | P Value: **0.0023** | | | | |  |  |
|  |  |  |  |  |  |  |  | OR: 0.86  (0.69 - 1.07) | OR: Ref |  |  |  |  |  |
|  |  |  |  |  |  |  |  | P Value: 0.1828 | | | | |  |  |
|  | **Sampling Frame** | | | | | **SES** | | **Utilization by SES Grouping (Number, %)** | | | | | | |
| **Study** | **Country** | **Data Source** | **Study Population^a^** | **Predictive Biomarker Test/ Biological and Precision Therapies Overall Utilization (Number, %)** | **Comparator** | **Unit** | **Measure** | **Lowest SES Group** | | **Highest SES Group** | | | **Utilization in Low SES (OR & 95% CI) ^b^** | **QA** |
| Webster *et al.* (2013)* | USA | Virtual Data Warehouse Tumour Registry Files | 7 CRN Sites Across the USA (Kaiser Permanente, Henry Ford Health System & Health Partners)  01/2004 - 12/2009  Stage III (Progressed to Distant Metastatic Disease) & IV  Colorectal Cancer  n = 1,188 | KRAS Tested  n = 428 (36.0) | Not KRAS Tested | Census Tract | Median Household Income | < $40K (Low)  95/316 (30.1) | $40K - $59K  142/405 (35.1) | $60K - $79K  90/231 (39.0) | ≥ $80K (High)  63/170 (37.1) |  | 0.73  (0.48 - 1.10) | 10 |
|  |  |  |  |  |  |  |  | P Value: **0.0024** | | | | |  |  |
|  |  |  |  |  |  |  |  | OR: 0.80  (0.50 - 1.40) | OR: Ref | OR: 1.30  (1.10 - 1.40) | OR: 1.20  (1.10 - 1.50) |  |  |  |
|  |  |  |  |  |  |  |  | P Value: 0.4753 | | | | |  |  |
|  |  |  |  |  |  | Census Tract | % with a High School Education | < 50% (Low)  4/16 (25.0) | 50 - 69%  36/99 (36.4) | 70 - 89%  197/580 (34.0) | ≥ 90% (High)  166/426 (39.0) |  | 0.52  (0.12 - 1.76) |  |
|  |  |  |  |  |  |  |  | P Value: **< 0.0001** | | | | |  |  |
|  |  |  |  |  |  |  |  | OR: 0.70  (0.10 - 3.30) | OR: 1.4  (1.00 - 1.80) | OR: Ref | OR: 1.20  (1.00 - 1.40) |  |  |  |
|  |  |  |  |  |  |  |  | P Value: 0.9924 |  |  |  |  |  |  |
| Enewold *et al.* (2017)*^d^ | USA | Hospital Medical Records, Contact with Treating Physicians and Others Involved Individual’s Care | NCI POC Study of SEER Patients (28% of US Population)  2011  Stage: Metastatic  Melanoma  n = 520^c^ | BRAF Test  n = 242 (46.5) | NR | Census Tract | Median Household Income | $15,769 - $41,350 (Low)  52/124 (41.9)^c^ | $41,351 - $55,155  61/128 (47.7)^c^ | $55,156 - $73,178  64/130 (49.2)^c^ | $73,179 - $163,393 (High)  63/133 (47.4)^c^ |  | 0.80  (0.48 - 1.35) | 4.5 |
|  |  |  |  |  |  |  |  | P Value: 0.70 | | | |  |  |  |
|  |  |  |  |  |  | Census Tract | % of Individuals Aged 25+ with at Least a High School Education | 28.30% - 77.28%  (Low)  50/126 (40.0)^c^ | 77.39% - 86.14%  60/126 (47.6)^c^ | 86.15% - 91.26%  65/133 (49.0)^c^ | 91.27% - 100.00%  (High)  65/130 (50.0)^c^ |  | 0.66  (0.39 - 1.11) |  |
|  |  |  |  |  |  |  |  | P Value: 0.46 | | | |  |  |  |
| Enewold & Thomas (2016)*^d^ | USA | Medical Records and Treating Physicians Contacted | NCI POC Study from SEER  2010  Stage IV  NSCLC  n = 764^c^ | EGFR Test  n = 152 (19.9) | NR | Census Tract | Median Income | < $43,000 (Low)  49/296 (16.5)^c^ | $43,000 - $62,000  46/248 (18.5)^c^ | >$62,000 (High)  55/220 (25.0)^c^ |  |  | 0.60  (0.38 - 0.94) | 5 |
|  |  |  |  |  |  |  |  | P Value: 0.39 | | | | |  |  |
|  | **Sampling Frame** | | | | | **SES** | | **Utilization by SES Grouping (Number, %)** | | | | | | |
| **Study** | **Country** | **Data Source** | **Study Population^a^** | **Predictive Biomarker Test/ Biological and Precision Therapies Overall Utilization (Number, %)** | **Comparator** | **Unit** | **Measure** | **Lowest SES Group** | | **Highest SES Group** | | | **Utilization in Low SES (OR & 95% CI) ^b^** | **QA** |
| Palazzo *et al.* (2019)*^d^ | USA | SEER - Medicare | 17 SEER Registries Comprising Approx 28% of US Population  2007 - 2011  Age ≥ 65  Stage IV  NSCLC  n = 9,900 | Genetic Test  n = 1,040  (10.5) | No Genetic Test | Eligibility for or Receipt of Low Income Subsidy for Medicare Part D | Income | Low Income (Low)  319/4,212  (7.6) | Not Low Income (High)  721/5,688  (12.7) |  |  |  | 0.56  (0.49 - 0.65) | 10 |
|  |  |  |  |  |  |  |  | P Values: **< 0.0001** | | | |  |  |  |
|  |  |  |  |  |  |  |  | OR: 0.78  (0.57 - 1.08) | OR: Ref |  |  |  |  |  |
|  |  |  |  |  |  |  |  | P Values: 0.1335 | | | |  |  |  |
|  |  |  |  |  |  | Census Tract | High Poverty Location | High Poverty (Low)  439/5,309  (8.3) | Not High Poverty (High)  554/4,202  (13.2) |  |  |  | 0.59  (0.52 - 0.68) |  |
|  |  |  |  |  |  |  |  | P Value: **< 0.0001** | | | | |  |  |
|  |  |  |  |  |  |  |  | OR: 0.82  (0.62 - 1.08) | OR: Ref |  |  |  |  |  |
|  |  |  |  |  |  |  |  | P Value: 0.1553 | | | | |  |  |
| Presley *et al.* (2018)* | USA | Flatiron Health Database | 250 Cancer Clinics with 1.5 Million Active Patients (Received Care at 1 of 191 Oncology Practices)  01/2011 - 07/2016  Stage IIIB - IV  Non-squamous NSCLC  n = 5,688 | Routine Test (EGFR and/or ALK)  n = 4,813 (84.6) | Broad Based Genomic Sequencing (Multigene Panel Testing More than 30 Genes) | Zip Code | Median Household Income | Q1 (Low)  484/555  (87.2) | Q2  637/732  (87.0) | Q3  927/1,090 (85.0) | Q4  1,037/1,214 (85.4) | Q5 (High)  1,612/1,969 (81.9) | 1.51  (1.14 - 2.01) | 6 |
|  |  |  |  |  |  |  |  | P Value**: < 0.001** | | | | |  |  |
|  | **Sampling Frame** | | | | | **SES** | | **Utilization by SES Grouping (Number, %)** | | | | | | |
| **Study** | **Country** | **Data Source** | **Study Population^a^** | **Predictive Biomarker Test/ Biological and Precision Therapies Overall Utilization (Number, %)** | **Comparator** | **Unit** | **Measure** | **Lowest SES Group** | | **Highest SES Group** | | | **Utilization in Low SES (OR & 95% CI) ^b^** | **QA** |
| **Breast Cancer: Biological & Precision Therapies** | | | | | | | | | | | | | | |
| Du *et al.* (2011)* | USA | SEER - Medicare | 16 USA Cancer Registries  1998 - 2005  Age ≥ 65  Stage I - IV & Unstaged  n = 47,806^c^ | Trastuzumab  & Anthracycline  n = 460 (1.0)  Trastuzumab & No Anthracycline  n = 414 (0.9) | Anthracycline/ Other Chemotherapy/  No Chemotherapy | Census Tract | % of Persons Living Below the Poverty Line | ≥ 12% (Low)  221/11,918 (1.9)^c^ | 6.63% - 11.99%  198/11,775 (1.7)^c^ | 3.63% - 6.62%  230/11,728 (2.0)^c^ | ≤3.62% (High)  222/11,855  (1.9)^c^ |  | 0.99  (0.82 - 1.20) | 5 |
| Vaz-Luis *et al.* (2015) | USA | SEER - Medicare | 28% of US Population  10/1998 - 12/2009  Age ≥ 66  Stage IV  White Women  N = 3,748 | HER2+ (Initiated Trastuzumab)  n = 347 (9.3) | HER2-  (No Trastuzumab) | Census Tract | Median Household Income | Q1 (Low)  64/742  (8.6)^e^ | Q2  78/944  (8.3)^e^ | Q3  96/1,015  (9.5)^e^ | Q4 (High)  109/1,047  (10.4)^e^ |  | 0.81  (0.58 - 1.14) | 5 |
|  |  |  |  |  |  | Census Tract | % With High School Diplomas | Q1 (Low)  65/788  (8.2)^e^ | Q2  87/931  (9.3)^e^ | Q3  99/983  (10.1)^e^ | Q4 (High)  96/1,046 (9.2)^e^ |  | 0.89  (0.63 - 1.25) |  |
| Reeder-Hayes *et al.* (2016)* | USA | SEER - Medicare | 28% of US Population  2010 - 2011  Age ≥ 66  Stage I - III  n = 1,362 | Trastuzumab Treated Users  n = 672 (49.3) | Untreated (No Trastuzumab) | Census Tract | Residents Below the Poverty Level | ≥ 20% (Low)  122/261 (46.7) | 10 - 19.99%  212/439 (48.3) | 5 - 9.99%  178/373 (47.7) | < 5% (High)  160/289 (55.4) |  | 0.71  (0.50 - 1.00) | 9.5 |
|  |  |  |  |  |  |  |  | P Value: 0.1397 | | | | |  |  |
|  |  |  |  |  |  |  |  | RR: 0.88  (0.76 - 1.02) | RR: 0.87  (0.77 - 0.99) | RR: 0.85  (0.75 - 0.97) | RR: Ref |  |  |  |
|  |  |  |  |  |  |  |  | P Value: 0.0965 | P Value: **0.0377** | P Value: **0.0155** |  | |  |  |
| Vaz- Luis *et al.* (2016) | USA | SEER - Medicare | 28% of US Population  2010 - 2011  Age ≥ 66  Stage ≥ Ib  n = 770^c^ | Trastuzumab  n = 428 (55.6) | No Trastuzumab | Census Tract | Median Household Income | Q1 (Low)  103/196  (52.6)^c^ | Q2  102/192  (53.1)^c^ | Q3  105/184  (57.1)^c^ | Q4 (High)  118/198  (59.6)^c^ |  | 0.75  (0.49 - 1.14) | 7.5 |
|  |  |  |  |  |  |  |  | P Value: 0.45 | | | | |  |  |
|  |  |  |  |  |  |  |  | OR (Non-Receipt):  Ref | OR (Non-Receipt): 1.22 (0.78 - 1.89) | OR (Non-Receipt):1.40 (0.82 - 2.20) | OR (Non-Receipt):1.60 (0.83 - 3.10) |  |  |  |
|  |  |  |  |  |  |  |  | P Value: 0.56 |  |  |  |  |  |  |
|  |  |  |  |  |  | Census Tract | High School Diploma Rate | Q1 (Low)  98/204  (48.0)^c^ | Q2  111/188  (59.0)^c^ | Q3  99/187  (52.9)^c^ | Q4 (High)  120/191  (62.8)^c^ |  | 0.55  (0.36 - 0.83) |  |
|  |  |  |  |  |  |  |  | P Value: **0.02** |  |  |  |  |  |  |
|  |  |  |  |  |  |  |  | OR (Non-Receipt):  Ref | OR (Non-Receipt):  2.32  (1.24 - 4.35) | OR (Non-Receipt):  1.21  (0.78 - 1.91) | OR (Non-Receipt):  1.68  (1.11 - 2.53) |  |  |  |
|  |  |  |  |  |  |  |  | P Value: **0.05** |  |  |  |  |  |  |
|  | **Sampling Frame** | | | | | **SES** | | **Utilization by SES Grouping (Number, %)** | | | | | | |
| **Study** | **Country** | **Data Source** | **Study Population^a^** | **Predictive Biomarker Test/ Biological and Precision Therapies Overall Utilization (Number, %)** | **Comparator** | **Unit** | **Measure** | **Lowest SES Group** | | **Highest SES Group** | | | **Utilization in Low SES (OR & 95% CI) ^b^** | **QA** |
| Tsai *et al.* (2017)* | USA | 3 SEER Registries, NYSCR, OCISS Linked to Healthcore Inc Claims | 5 US States  01/ 2006 - 12/2011  Age < 64  Stage I - III  HER2+  n = 934 | Trastuzumab  n = 680 (72.8) | NR | Census Tract | Median Household Income | Q1 (Low)  104/140  (74.3) | Q2  121/176  (68.8) | Q3  139/185  (75.1) | Q4  151/204  (74.0) | Q5 (High)  155/216  (71.8) | 1.14  (0.69 -1.90)  1.07  (0.60 -1.89) | 6 |
|  |  |  |  |  |  | Census Tract | % Completing College | < 20%  127/169  (75.1) | 20% to < 30%  289/406  (71.2) | 30% to < 40%  166/227  (73.1) | ≥ 40%  88/119  (73.9) | Overall P Value: 0.85 |  |  |
|  |  |  |  |  |  |  |  | OR: Ref | OR: 0.82 (0.50 - 1.35) | OR: 0.84  (0.48 - 1.45) | OR: 0.95 (0.51 - 1.79) |  |  |  |
|  |  |  |  |  |  |  |  |  | P Value: 0.44 | P Value: 0.53 | P Value: 0.89 |  |  |  |
| Freedman *et al.*(2013)* | USA | NCCN Breast Cancer Outcomes Database | 8 US Centers  09/2005 - 12/2008  Stage I - III  n = 1,109 | Trastuzumab  n = 925 (83.0) | NR | Patient Reported | Educational Attainment | < High School (Low)  46/54  (85.2) | High School Degree  131/161  (81.4) | Some College  182/218  (83.5) | College/  Graduate Degree  (High)  341/404  (84.4) |  | 1.06  (0.47 - 2.73) | 8 |
|  |  |  |  |  |  |  |  | P Value: 0.911 | | | |  |  |  |
|  |  |  |  |  |  |  |  | OR 1.58  (0.98 - 2.52) | OR: 0.84 (0.58 - 1.23) | OR: 0.94 (0.67 - 1.32) | OR: Ref |  |  |  |
|  |  |  |  |  |  | Patient Reported | Employment | Unemployed (Low)  61/69  (88.4) | Retired  110/153  (71.9) | Homemaker  148/178  (83.1) | Employed/  Student (High)  491/577  (85.1) |  | 1.34  (0.61 - 3.35) |  |
|  |  |  |  |  |  |  |  | P Value: **0.002** | | | | |  |  |
|  |  |  |  |  |  |  |  | OR = 1.11  (0.56 - 2.21) | OR: 1.37 (0.96 - 1.94) | OR: 0.89 (0.69 - 1.15) | OR: Ref |  |  |  |
| Haas *et al.* (2011)* | USA | Aetna Medical Records | US Women Receiving Aetna Health Coverage  07/2006 - 06/2007  Age: 35 - 65  Stage I - III  HER2+ Test Result  n = 137 | Trastuzumab  n = 79 (57.7) | NR | Small Area Estimation | Annual Household Income | < $40,000 (Low)  27/34  (79.4) | $40,000 - $74,999  22/47  (46.8) | $75,000 - $124,999  16/27  (59.3) | ≥$125,000 (High)  11/23  (47.8) |  | 4.21  (1.14 - 16.04) | 8.5 |
|  |  |  |  |  |  |  |  | P Value: **0.02** | | | | |  |  |
|  |  |  |  |  |  |  |  | OR: 4.43  (1.22 - 16.04) | OR: 1.01  (0.34 - 3.00) | OR: 1.61 (0.47 - 5.51) | OR: Ref |  |  |  |
|  | **Sampling Frame** | | | | | **SES** | | **Utilization by SES Grouping (Number, %)** | | | | | | |
| **Study** | **Country** | **Data Source** | **Study Population^a^** | **Predictive Biomarker Test /Biological and Precision Therapies Overall Utilization (Number, %)** | **Comparator** | **Unit** | **Measure** | **Lowest SES Group** | | **Highest SES Group** | | | **Utilization in Low SES (OR & 95% CI) ^b^** | **QA** |
| Goldhar *et al.* (2016) | Canada | OCR Linked to RPBD, CIHI-DAD, OHIP, ODB, NDFP & NACRS | Ontario  2003 - 2009  Stage I - III  n = 19,074 | Trastuzumab and Chemotherapy  n = 3,371 (17.7) | Chemotherapy Alone | Postal Code | Average Household Income | Q1 (Low)  564/3,029  (18.6) | Q2  625/3,541  (17.7) | Q3  667/3,863  (17.3) | Q4  722/4,191  (17.2) | Q5 (High)  781/4,385  (17.8) | 1.06  (0.93 - 1.19) | 6 |
|  |  |  |  |  |  |  |  | P Value: 0.57 | | | | |  |  |
| Kumachev *et al.* (2016)* | Canada | OCR Linked to RPBD, CIHI-DAD, OHIP, NDFP, NACRS & ODB | Ontario  01/2004 - 12/2009  Age > 18  Stage I - III  n = 33,056 | Trastuzumab  n = 3,391  (10.3) | NR | Postal Code | Average Neighborhood Income | Q1 (Low)  577/5,890 (9.8) | Q2  645/6,448  (10.0) | Q3  662/6,362  (10.4) | Q4  725/6,906  (10.5) | Q5 (High)  782/7,450  (10.5) | 0.93  (0.83 - 1.04) | 4.5 |
|  |  |  |  |  |  |  |  | P Value: 0.62 | | | | |  |  |
| ***Thavendiranathan et al.***  (***2016**** & 2018) | Canada | OCR Linked to RPBD, CIHI-DAD, OHIP, NACRS, NDFP, CALR & ICES | 14 Ontario Cancer Centers  07/2007 - 12/2012  Age > 18  Stage I - III  n = 18,540 | Trastuzumab Without Anthracycline/  n = 832 (4.5)  Sequential Therapy (Anthracycline followed by Trastuzumab)  n = 3,250 (17.5) | Anthracycline Without Trastuzumab/  Other Chemotherapy | Postal Code | Median Income | Q1 (Low)  658/3,088  (21.3) | Q2  775/3,454  (22.4) | Q3  812/3,658  (22.2) | Q4  952/4,102  (23.2) | Q5 (High)  885/4,238  (20.9) | 1.03  (0.91 - 1.15) | 5 |
| Li *et al.* (2018)* | China | Hospital Records, Telephone Conversations & Other Means | 155 Hospitals (29 Chinese Provinces)  07/2013 - 06/2014  Age ≥ 18  Stage I - III  n = 4,994 | Trastuzumab  n = 1,487  (29.8) | Non-Trastuzumab | NR | Household Income | < 10,000 Yuan (Low)  12/54 (22.2) | 10,000 - 30,000 Yuan  55/276 (19.9) | 30,000 - 50,000 Yuan  82/284 (28.9) | > 50,000 Yuan (High)  168/357(47.1) |  | 0.32  (0.15 - 0.65) | 6 |
|  |  |  |  |  |  |  |  | P Value: **< 0.001** | | | | |  |  |
|  |  |  |  |  |  | NR | Education Level | Primary School or Lower (Low)  112/469  (23.9) | High School  238/738  (32.2) | College or Higher (High)  133/266  (50.0) |  |  | 0.31  (0.22 - 0.44) |  |
|  |  |  |  |  |  |  |  | P Value: **< 0.001** | | | | |  |  |
| Shih *et al.* (2009)^f^ | USA | NCDB | Captures 75% of Newly Diagnosed Cancer Cases  1998 - 2004  Stage: IV  n = 42,804 | Immunotherapy  n = 1,723 (4.0) | NR | Zip Code | % Without a High School Degree | 29%+  OR: 0.98  (0.67 - 1.44)^g^ | 20% - 28.9%  OR: 0.77  (0.55 - 1.08)^g^ | 14% - 19.9%  OR: 0.92 (0.68 - 1.24)^g^ | < 14%  OR: Ref^g^ |  | ^h^ | 7 |
|  |  |  |  |  |  | Zip Code | Median Household Income | < $30,000  OR: Ref^g^ | $30,000 - $34,999  OR: 1.42 (0.99 - 2.06)^g^ | $35,000 - $45,999  OR: 1.11 (0.77 - 1.62)^g^ | $46,000+  OR:1.05  (0.71 - 1.55)^g^ |  |  |  |
|  | **Sampling Frame** | | | | | **SES** | | **Utilization by SES Grouping (Number, %)** | | | | | | |
| **Study** | **Country** | **Data Source** | **Study Population^a^** | **Predictive Biomarker Test/ Biological and Precision Therapies Overall Utilization (Number, %)** | **Comparator** | **Unit** | **Measure** | **Lowest SES Group** | | **Highest SES Group** | | | **Utilization in Low SES (OR & 95% CI) ^b^** | **QA** |
| **Non-Small-Cell Lung Cancer: Biological & Precision Therapies** | | | | | | | | | | | | | | |
| Zhu *et al.* (2012) | USA | SEER - Medicare | 17 US Registries (28% of US Population)  2006 - 2007  Age ≥ 65  Stage IIB - IV  Non-squamous  n = 1,500  n (PSM) = 636 | Bevacizumab with Carboplatin and Paclitaxel  n = 318 (21.2)  n (PSM) = 318 (50.0) | Carboplatin & Paclitaxel | Census Tract | Median Household Income | Q1 (Low)  60/300  (20.0) | Q2  58/300  (19.3) | Q3  63/300  (21.0) | Q4  58/300  (19.3) | Q5 (High)  79/300  (26.3) | 0.70  (0.47 - 1.04) | 8 |
|  |  |  |  |  |  |  |  | P Value: 0.20 | | | | |  |  |
|  |  |  |  |  |  |  |  | PSM:  60/123  (48.8) | PSM:  58/106  (54.7) | PSM:  63/128  (49.2) | PSM:  58/116  (50.0) | PSM:  79/163 (48.5) |  |  |
|  |  |  |  |  |  |  |  | P Value: 0.88 | | | | |  |  |
|  |  |  |  |  |  | Census Tract | % of Persons Older than Age 25 with Some College Education | Q1 (Low)  66/300 (22.0) | Q2  58/302 (19.2) | Q3  62/300 (20.7) | Q4  57/298 (19.1) | Q5 (High)  75/300 (25.0) | 0.85  (0.57 - 1.26) |  |
|  |  |  |  |  |  |  |  | P Value: 0.38 | | | | |  |  |
|  |  |  |  |  |  |  |  | PSM:  66/147  (44.9) | PSM:  58/104  (55.8) | PSM:  62/117  (53.0) | PSM:  57/125  (45.6) | PSM:  75/143 (52.4) |  |  |
|  |  |  |  |  |  |  |  | P Value: 0.33 | | | | |  |  |
| Langer *et al.* (2014)* | USA | SEER - Medicare | 20 Geographic Areas  (29% of US Population)  2006 - 2009  Age ≥ 65  Stage IIIB - IV  Non-squamous  n = 1,706 | Bevacizumab with Carboplatin & Paclitaxel  n = 592 (34.7) | Carboplatin & Paclitaxel | Census Tract | Median Household Income | Q1(Low)  96/340  (28.2) | Q2  123/341  (36.1) | Q3  129/343  (37.6) | Q4  113/340  (33.2) | Q5 (High)  131/342 (38.3) | 0.63  (0.45 - 0.88) | 6 |
|  |  |  |  |  |  |  |  | P Value: **0.039** | | | | |  |  |
|  |  |  |  |  |  | Census Tract | % of Persons Older than 25 Years with Some College Education | Q1 (Low)  117/340  (34.4) | Q2  111/341  (32.6) | Q3  111/343  (32.4) | Q4  119/341  (34.9) | Q5 (High)  134/341 (39.3) | 0.81  (0.59 - 1.12) |  |
|  |  |  |  |  |  |  |  | P Value: 0.317 | | | | |  |  |
| ***Ritzwoller et al. (2014)***, Delate *et al*. (2014) & Carroll *et al.* (2015) | USA | CRN’s Virtual Data Warehouse | 4 US HMOs  2005 - 2010  Age ≥ 21  Stage IIIB - IV  Non-squamous  n = 1,109 | Bevacizumab with Carboplatin & Paclitaxel  n = 198 (17.9) | Carboplatin & Paclitaxel | Census Tract | % College Educated | Q1 (Low)  30/224  (13.4) | Q2  50/226  (22.1) | Q3  38/224  (17.0) | Q4  42/217  (19.4) | Q5 (High)  38/218 (17.4) | 0.73  (0.42 - 1.27) | 6.5 |
|  |  |  |  |  |  |  |  | P Value: 0.17 | | | | |  |  |
|  |  |  |  |  |  | Census Tract | Median Family Income | Q1 (Low)  36/219  (16.4) | Q2  44/228  (19.3) | Q3  47/221  (21.3) | Q4  38/224  (17.0) | Q5 (High)  33/217 (15.2) | 1.10  (0.63 - 1.90) |  |
|  |  |  |  |  |  |  |  | P Value: 0.47 | | | | |  |  |
|  | **Sampling Frame** | | | | | **SES** | | **Utilization by SES Grouping (Number, %)** | | | | | | |
| **Study** | **Country** | **Data Source** | **Study Population^a^** | **Predictive Biomarker Test/ Biological and Precision Therapies Overall Utilization (Number, %)** | **Comparator** | **Unit** | **Measure** | **Lowest SES Group** | | **Highest SES Group** | | | **Utilization in Low SES (OR & 95% CI) ^b^** | **QA** |
| Menter *et al.* (2016)* | USA | CRN’s Virtual Data Warehouse | 4 Kaiser Permanente Regions  01/2005 - 12/2011  Age ≥ 21  Stage IIIB - IV  Non-squamous  n = 1,813  n (PSM) = 632 | Bevacizumab with Carboplatin & Paclitaxel  n = 348 (19.2)  n (PSM) = 122 (19.3) | Carboplatin & Paclitaxel | Census | Education | Rank 1 (Low)  66/329  (20.1) | Rank 2  51/338  (15.1) | Rank 3  76/374  (20.3) | Rank 4  67/382  (17.5) | Rank 5 (High)  88/390 (22.6) | 0.86  (0.59 - 1.25) | 5.5 |
|  |  |  |  |  |  |  |  | PSM:  14/97  (14.4) | PSM:  33/122  (27.0) | PSM:  23/115  (20.0) | PSM:  28/166  (16.9) | PSM:  24/114 (21.1) | 0.63  (0.28 - 1.38) |  |
| Maguire *et al.* (2019a) & ***Maguire et al. (2019b)*****^i^* | USA | CCR  Affiliated with SEER | California, USA  2012 - 2014  Age ≥ 20  Stage IV  n = 17,254 | Bevacizumab Based (Alone or in Combination with Chemotherapy)  n = 530 (3.1)  Pemetrexed & Bevacizumab Based (Together or with a Platinum Agent)  n = 635 (3.7) | Platinum Doublets/  Pemetrexed Based/  Single Agents/  TKIs/  Chemotherapy/  No Treatment/ Unknown | Census Block | SES (Aggregate Measure of Education, Occupation, Unemployment, Household Income, Poverty, Rent & Home Price) | Q1 (Low)  152/2,888 (5.3) | Q2  204/3,530 (5.8) | Q3  237/3,703 (6.4) | Q4  272/3,771 (7.2) | Q5 (High)  300/3,362 (8.9) | 0.57  (0.46 - 0.70) | 8.5 |
|  |  |  |  |  |  |  |  | Bevacizumab Based  OR: 0.60  (0.43 - 0.85) | Bevacizumab Based  OR: 0.71  (0.52 - 0.97) | Bevacizumab Based  OR: 0.82  (0.61 - 1.09) | Bevacizumab Based  OR: 0.93  (0.70 - 1.23) |  |  |  |
|  |  |  |  |  |  |  |  | Pemetrexed & Bevacizumab Based  OR: 0.40  (0.29 - 0.54) | Pemetrexed & Bevacizumab Based  OR: 0.47  (0.36 - 0.62) | Pemetrexed & Bevacizumab Based  OR: 0.50  (0.39 - 0.65) | Pemetrexed & Bevacizumab Based  OR: 0.62 (0.48 - 0.79) |  |  |  |
|  |  |  |  | TKIs *^i^*  n = 1,711 (9.9) | Platinum Doublets/  Pemetrexed Based/ Bevacizumab Based/  Pemetrexed & Bevacizumab/  Single Agents/  Chemotherapy/  No Treatment/ Unknown | Census Block | SES (Aggregate Measure of Education, Occupation, Unemployment, Household Income, Poverty, Rent & Home Price) | Q1 (Low)  159/2,888 (5.5) | Q2  287/3,530 (8.1) | Q3  340/3,703 (9.2) | Q4  412/3,771 (10.9) | Q5 (High)  513/3,362 (15.3) | 0.32  (0.27 - 0.39) |  |
|  |  |  |  |  |  |  |  | OR: 0.30  (0.24 - 0.37) | OR: 0.51 (0.42 - 0.62) | OR: 0.53 (0.44 - 0.63) | OR: 0.66 (0.55 - 0.79) | OR: Ref |  |  |
|  | **Sampling Frame** | | | | | **SES** | | **Utilization by SES Grouping (Number, %)** | | | | | | |
| **Study** | **Country** | **Data Source** | **Study Population^a^** | **Predictive Biomarker Test/ Biological and Precision Therapies Overall Utilization (Number, %)** | **Comparator** | **Unit** | **Measure** | **Lowest SES Group** | | **Highest SES Group** | | | **Utilization in Low SES (OR & 95% CI) ^b^** | **QA** |
| Palazzo e*t al*. (2019)* ^d^ | USA | SEER - Medicare | 17 US Registries  (28% of US Population)  2007 - 2011  Age ≥ 65  Stage IV  Had Genetic Test  n = 1,040 | Erlotinib & had Genetic Test  n = 250  (24.0) | NR | Eligibility for or Receipt of Low Income Subsidy for Medicare Part D | Income Level | Low Income (Low)  73/319 (22.9) | Not Low Income (High)  177/721 (24.5) |  |  |  | 0.91  (0.66 - 1.23) | 9 |
|  |  |  |  |  |  |  |  | P Value: **0.0131** | |  |  |  |  |  |
|  |  |  |  |  |  |  |  | OR: 0.32  (0.13 - 0.79) | OR: Ref |  |  |  |  |  |
|  |  |  |  |  |  |  |  | P Value: **0.0131** | |  |  |  |  |  |
|  |  |  |  |  |  | Census Tract | Residence in a High Poverty Location | High Poverty  (Low)  102/439 (23.2) | Not High Poverty (High)  136/554 (24.5) |  |  |  | 0.93  (0.69 - 1.26) |  |
|  |  |  |  |  |  |  |  | P Value: **0.0002** | |  |  |  |  |  |
|  |  |  |  |  |  |  |  | OR: 1.12  (0.58 - 2.17) | OR: Ref |  |  |  |  |  |
|  |  |  |  |  |  |  |  | P Value: 0.7304 | |  |  |  |  |  |
| Enewold & Thomas (2016)^d^ | USA | SEER Medical Records & Querying Treating Physicians | NCI POC Study of SEER Patients  2010  Age ≥ 20  Stage IV  n = 764^c^ | Erlotinib  n = 70  (9.2) | NR | Census Tract | Median Income | < $43,000 (Low)  18/296 (6.0)^c^ | $43,000 - $62,000  17/248 (7.0)^c^ | > $62,000 (High)  32/220 (14.7)^c^ |  |  | 0.38  (0.20 - 0.72) | 6 |
|  |  |  |  |  |  |  |  | P Value: 0.11 | | |  |  |  |  |
|  |  |  |  |  |  | Census Tract | % With a High School Education | < 77% (Low)  29/212 (13.9)^c^ | 77 - 89%  13/221 (5.9)^c^ | > 89% (High)  24/331 (7.1)^c^ |  |  | 2.03  (1.10 - 3.75) |  |
|  |  |  |  |  |  |  |  | P Value: 0.15 | | |  |  |  |  |
| Verma *et al.* (2019)* | USA | NCDB | 70% of US Malignancies Annually  2004 - 2015  Age ≥ 18  Stage IV  n = 504,447 | Immunotherapy  n = 11,420 (2.3) | No Immunotherapy | NR | Income  (US $/Year) | < $63,000 (Low)  7,886/360,070 (2.2) | ≥ $63,000 (High)  3,467/134,988 (2.6) |  |  |  | 0.85  (0.82 - 0.88) | 8 |
|  |  |  |  |  |  |  |  | P Value: **< 0.001** | |  |  |  |  |  |
|  |  |  |  |  |  |  |  | OR: Ref | OR: 0.993 (0.945 -1.044) |  |  |  |  |  |
|  |  |  |  |  |  |  |  | P Value: 0.795 | |  |  |  |  |  |
|  |  |  |  |  |  | Zip Code | Education  (% With a High School Diploma) | < 80% (Low)  4,339/215,197 (2.0) | ≥ 80% (High)  6,734/270,482 (2.5) |  |  |  | 0.81  (0.78 - 0.84) |  |
|  |  |  |  |  |  |  |  | P Value: **< 0.001** | |  |  |  |  |  |
|  |  |  |  |  |  |  |  | OR: Ref | OR: 1.140 (1.087 -1.197) |  |  |  |  |  |
|  |  |  |  |  |  |  |  | P Value: **< 0.001** | |  |  |  |  |  |
|  | **Sampling Frame** | | | | | **SES** | | **Utilization by SES Grouping (Number, %)** | | | | | | |
| **Study** | **Country** | **Data Source** | **Study Population^a^** | **Predictive Biomarker Test/ Biological and Precision Therapies Overall Utilization (Number, %)** | **Comparator** | **Unit** | **Measure** | **Lowest SES Group** | | **Highest SES Group** | | | **Utilization in Low SES (OR & 95% CI) ^b^** | **QA** |
| Lairson *et al.* (2015)* | USA | SEER - Medicare | 17 US Registries  01/2006 - 12/2009  Age 65 - 94  Stage IIIB - IV  n (PSM) = 4,884 | Targeted Therapy & Platinum Based Therapy  n (PSM) = 1,628  (33.3) | Platinum Based Chemotherapy/  No Chemotherapy | NR | Poverty Level | 1^st^ (Low)  290/863  (33.6) | 2^nd^  400/1,256 (31.8) | 3^rd^  425/1,242 (34.2) | 4^th^ (High)  513/1,523 (33.7) |  | 1.00  (0.83 - 1.19) | 6 |
| **Hepatobiliary Cancer: Biological & Precision Therapies** | | | | | | | | | | | | | | |
| Sahara *et al.* (2019)* | USA | NCDB | 1,500 Hospitals that Represent 70% of New Oncology Cases  01/2004 - 12/2015  Stage: I - IV  n = 249,913  Hepatobiliary | Immunotherapy  n = 585 (0.2) | No Immunotherapy | NR | Median Income | < $30,000 (Low)  79/40,313 (0.2) | $ 30,000 - $35,999  86/45,053 (0.2) | $36,000 - $45,999  157/67,104 (0.2) | ≥ $46,000 (High)  242/87,867 (0.3) |  | 0.71  (0.54 - 0.92) | 8 |
|  |  |  |  |  |  |  |  | P Value: **0.006** | | | |  |  |  |
|  |  |  |  |  |  |  |  | Multivariate OR: Ref | Multivariate OR: 1.01 (0.74 - 1.38) | Multivariate OR: 1.23 (0.93 - 1.62) | Multivariate OR: 1.43 (1.11 - 1.87) |  |  |  |
| Sanoff *et al.* (2016)* | USA | SEER - Medicare | 28% of US Population  2008 - 2011  Stage: Advanced Hepatocellular Cancer  n =1,532 | Sorafenib  n = 422 (27.5) | No Treatment | Census Tract | % Below the Poverty Line | Q1 (Low)  104/335 (31.0) | Q2  99/368 (26.9) | Q3  109/414(26.3) | Q4 (High)  108/397(27.2) |  | 1.20  (0.86 - 1.68) | 6 |
| Parsons *et al.* (2017) | USA | SEER | NCI POC Study of SEER Patients  2007 & 2012  BCLC Stage C  Hepatocellular Cancer  n = 550 | Sorafenib  n = 186 (33.8) | NR | Census Tract | Median Income Per Year for the Individual’s Census Tract | ≤ $50,000 (Low)  62/264 (23.5) | > $50,000 (High)  96/286 (33.6) |  |  |  | 0.61  (0.41 - 0.90) | 6.5 |
|  |  |  |  |  |  |  |  | P Value: 0.06 | |  |  |  |  |  |
|  |  |  |  |  |  |  |  | OR: Ref | OR: 1.50 (0.89 - 2.55) |  |  |  |  |  |
|  |  |  |  |  |  |  |  | P Value: > 0.05 | |  |  |  |  |  |
|  | **Sampling Frame** | | | | | **SES** | | **Utilization by SES Grouping (Number, %)** | | | | | | |
| **Study** | **Country** | **Data Source** | **Study Population^a^** | **Predictive Biomarker Test/ Biological and Precision Therapies Overall Utilization (Number, %)** | **Comparator** | **Unit** | **Measure** | **Lowest SES Group** | | **Highest SES Group** | | | **Utilization in Low SES (OR & 95% CI) ^b^** | **QA** |
| Sarpel *et al.* (2018)* | USA | Electronic Medical Records | Urban, Tertiary Academic Healthcare and Leading Referral Center for Liver Diseases  2007 - 2013  Stage: Early which Progressed to Advanced (BCLC Stage C)  Hepatocellular Cancer  n = 959 | Sorafenib  n = 352 (36.7) | NR | Zip Code | Education  (% with a Bachelors Degree) | < 24.3% (Low)  115/326 (35.3) | 24.3% -29.75%  82/261 (31.4) | 29.75% - 42.3%  82/192 (42.7) | ≥ 42.3% (High)  73/180 (40.6) |  | 0.80  (0.54 - 1.18) | 8.5 |
|  |  |  |  |  |  | Zip Code | Estimated Annual Median Income | < $37,309 (Low)  68/232 (29.3) | $37,309 - $55,965  116/368 (31.5) | $55,965 - $ 82,814  84/215 (39.1) | ≥ $82,814 (High)  84/144 (58.3) |  | 0.30  (0.19 - 0.47) |  |
|  |  |  |  |  |  |  |  | OR (Higher vs Lower): 2.05 (1.19 - 3.54) | | | |  |  |  |
|  |  |  |  |  |  |  |  | P Value: **< 0.01** | | | |  |  |  |
| **Melanoma: Biological & Precision Therapies** | | | | | | | | | | | | | | |
| Enewold *et al.* (2017)*^d^ | USA | SEER Hospital Medical Records, Contact with Treating Physicians and Others Involved Individual’s Care | NCI POC Study of SEER Patients  2011  Age ≥ 20  Metastatic Melanoma  n = 520^c^ | Ipilimumab  n = 109 (21.0) | NR | Census Tract | Median Household Income | $ 15,769 - $41,350  (Low)  22/124 (17.5)^c^ | $41,351 - $55,155  21/128 (16.2)^c^ | $55,156 - $73,178  30/130 (23.4)^c^ | $73,179 - $163,393 (High)  33/133 (25.1)^c^ |  | 0.65  (0.34 - 1.25) | 5 |
|  |  |  |  |  |  |  |  | P Value: 0.22 | | | |  |  |  |
|  |  |  |  |  |  | Census Tract | % of Individuals Aged 25+ with at Least a High School Education | 28.30% - 77.28%  (Low)  24/126 (19.1)^c^ | 77.39% - 86.14%  22/126 (17.5)^c^ | 86.15% - 91.26%  27/133 (20.5)^c^ | 91.27% - 100.00% (High)  33/130 (25.2)^c^ |  | 0.69  (0.36 - 1.31) |  |
|  |  |  |  |  |  |  |  | P Value: 0.41 | | | |  |  |  |
|  | **Sampling Frame** | | | | | **SES** | | **Utilization by SES Grouping (Number, %)** | | | | | | |
| **Study** | **Country** | **Data Source** | **Study Population^a^** | **Predictive Biomarker Test/ Biological and Precision Therapies Overall Utilization (Number, %)** | **Comparator** | **Unit** | **Measure** | **Lowest SES Group** | | **Highest SES Group** | | | **Utilization in Low SES (OR & 95% CI) ^b^** | **QA** |
| Al-Qurayshi *et al.* (2018)* | USA | NCDB | Captures 70% of Newly Diagnosed Malignancies  2004 - 2012  Age ≥ 18  Stage III  Cutaneous Melanoma  n = 6,165^c^ | Immunotherapy & Surgery  n = 1,854 (30.1) | Surgery Only | Zip Codes | Community Household Income | Q1 (Low)  202/746 (27.1)^c^ | Q2  386/1,307 (29.5)^c^ | Q3  523/1,702 (30.7)^c^ | Q4 (High)  743/2,411 (30.8)^c^ |  | 0.83  (0.69- 1.01) | 6.5 |
|  |  |  |  |  |  |  |  | P Value: 0.22 | | | |  |  |  |
|  |  |  |  |  |  |  |  | OR: 1.27  (0.99 - 1.62) | OR: 1.18  (0.98 - 1.43) | OR: 1.13 (0.96 - 1.33) | OR: Ref |  |  |  |
|  |  |  |  |  |  |  |  | P Value: **0.06** | P Value: 0.08 | P Value: 0.13 |  |  |  |  |
|  |  |  |  |  |  | Zip Codes | High School Graduation Rate | Q1 (Low)  178/758 (23.5)^c^ | Q2  404/1,412 (28.6)^c^ | Q3  595/1,893 (31.4)^c^ | Q4 (High)  677/2,108 (32.1)^c^ |  | 0.65  (0.53 - 0.79) |  |
|  |  |  |  |  |  |  |  | P Value: **< 0.001** | | | |  |  |  |
|  |  |  |  |  |  |  |  | OR: 0.59  (0.45 - 0.76) | OR: 0.80 (0.66 - 0.97) | OR: 0.94 (0.80 - 1.10) | OR: Ref |  |  |  |
|  |  |  |  |  |  |  |  | P Value: **<0.001** | P Value: **0.02** | P Value: 0.43 |  |  |  |  |
| Haque *et al.* (2019)* | USA | NCDB | Captures 70% of Newly Diagnosed Malignancies  2004 - 2014  Age ≥ 18  Metastatic Melanoma  n = 15,941 | Immunotherapy  n = 2,448 (15.4) | No Immunotherapy | Zip Code | Median Annual Income | < $63,000 (Low)  1,482/10,418 (14.2) | ≥ $63,000 (High)  929/5,202 (17.9) |  |  |  | 0.76  (0.70 - 0.84) | 8.5 |
|  |  |  |  |  |  |  |  | P Value: **< 0.001** | |  |  |  |  |  |
|  |  |  |  |  |  |  |  | OR: Ref | OR: 1.233 (1.118 - 1.360) |  |  |  |  |  |
|  |  |  |  |  |  |  |  | P Value: **< 0.001** | |  |  |  |  |  |
| **Colorectal Cancer: Biological & Precision Therpaies** | | | | | | | | | | | | | | |
| Fu *et al.* (2014)* | USA | SEER - Medicare | 17 US Registries  (26% of US Population)  01/2005 - 12/2009  Age ≥ 65  Stage: IV & Early CRC with Progression or Recurrence  n = 8,645^c^ | Bevacizumab with Chemotherapy  n = 4,502 (52.1) | No Bevacizumab | Census Tract  Census Tract | Median Household Income | Q1 (Low)  1,062/2,083 (51.0)^c^ | Q2  1,112/2,101 (53.0)^c^ | Q3  1,130/2,161 (52.3)^c^ | Q4 (High)  1,175/2,265 (52.0)^c^ |  | 0.96  (0.86 - 1.09) | 8.5 |
|  |  |  |  |  |  |  |  | P Value: 0.66 | | | | |  |  |
|  |  |  |  |  |  |  |  | Q1  OR: Ref |  |  | Q4  OR: 1.20 (1.03 - 1.40) |  |  |  |
|  |  |  |  |  |  |  |  | P Value: **0.021** | | | | |  |  |
|  |  |  |  |  |  |  | % of Adults with Less than a High School Education | Q1 (Low)  1,126/2,196 (51.3)^c^ | Q2  1,126/2,187 (51.5)^c^ | Q3  1,071/2,075 (51.6)^c^ | Q4 (High)  1,162/2,153 (54.0)^c^ |  | 0.90  (0.80 - 1.01) |  |
|  |  |  |  |  |  |  |  | P Value: 0.20 | | | | |  |  |
|  | **Sampling Frame** | | | | | **SES** | | **Utilization by SES Grouping (Number, %)** | | | | | | |
| **Study** | **Country** | **Data Source** | **Study Population^a^** | **Predictive Biomarker Test/ Biological and Precision Therapies Overall Utilization (Number, %)** | **Comparator** | **Unit** | **Measure** | **Lowest SES Group** | | **Highest SES Group** | | | **Utilization in Low SES (OR & 95% CI) ^b^** | **QA** |
| Meyerhardt *et al.* (2012) | USA | SEER - Medicare | 16 US Registries  (26% of US Population)  2002 - 2007  CRC  Age ≥ 65  Stage IV  n = 2,526 | Bevacizumab with Combination Chemotherapy  n = 903 (35.7) | Combination Chemotherapy Without Bevacizumab | Zip Code | Median Income | Q4 (Low)  214/631 (33.9) | Q3  229/631 (36.3) | Q2  229/631 (36.3) | Q1 (High)  231/632 (36.6) |  | 0.89  (0.70 - 1.13) | 5.5 |
| Cen *et al.* (2012)* | USA | SEER - Medicare | 17 US Registries  (26% of US Population)  2003 - 2005  Age ≥ 65  Stage I - IV  CRC  n = 46,692^c^ | Chemotherapy Containing Bevacizumab Based Regimens  n =1,306 (2.8) | No Chemotherapy/ Other Chemotherapy/  5-FU Alone/ Oxaliplatin Based Chemotherapy Regimens | Census Tract | % of Residents Below the Poverty Line | 4^th^ (Low)  272/11,643 (2.3)^c^ | 3^rd^  337/11,669 (2.9)^c^ | 2^nd^  336/11,636 (2.9)^c^ | 1^st^ (High)  360/11,662 (3.1)^c^ |  | 0.75  (0.64 - 0.88) | 4 |
| Parikh *et al.* (2016)* | USA | SEER - Medicare | 16 US Registries  (28% of US Population)  01/2004 - 12/2009  Age ≥ 65  Stage IV  CRC  n = 4,418 | First Line Chemotherapy with a Targeted Biologic  n = 2,077 (47.0) | First Line Chemotherapy Treatment | Zip Code | % Below the Poverty Line | 1^st^ (Low)  457/989  (46.2) | 2^nd^  504/1,070 (47.1) | 3^rd^  549/1,139 (48.2) | 4^th^ (High)  567/1,220 (46.5) |  | 0.99  (0.83 - 1.17) | 7 |
|  |  |  |  |  |  |  |  | OR: Ref | OR: 1.07 (0.89 - 1.29) | OR: 1.13 (0.93 - 1.37) | OR: 1.10 (0.89 - 1.35) |  |  |  |
| Neugut *et al.* (2012) | USA | SEER - Medicare | 26% of US  01/2005 - 12/2005  Colon Cancer  Age ≥ 65  Stage IV  n = 859 | Chemotherapy with Bevacizumab  n = 310 (36.1) | Chemotherapy without Bevacizumab | Census Tract | SES Score based on education, poverty and income | 1^st^ (Low)  OR: Ref | 2^nd^  OR: 1.98 (0.78 - 5.05) | 3^rd^  OR: 1.23 (0.50 - 3.01) | 4^th^  OR: 1.24 (0.48 - 3.26) | 5^th^ (High)  OR: 2.30 (0.86 - 6.18) | ^h^ | 6.5 |
| Raab *et al.* (2019)* | USA | SEER - Medicare | 26% of US Population  01/2005 -  12/2013  Age ≥ 65  Stage IV  Colon Cancer  n = 3,785 | Bevacizumab in Conjunction with Chemotherapy  n = 2,352 (62.1) | Did Not Receive Bevacizumab in Conjunction with Chemotherapy | Census Tract | SES Rank (Education Level, Poverty Level & Income Combined Score) | Rank 0  (Low)  409/632  (64.7) | Rank 1  484/747  (64.8) | Rank 2  366/619  (59.1) | Rank 3  608/994  (61.2) | Rank 4 (High)  485/792 (61.2) | 1.16  (0.93 - 1.45) | 9 |
|  |  |  |  |  |  |  |  | P Value: > 0.05 | | | | |  |  |
|  |  |  |  |  |  |  |  | OR: Ref | OR:1.00  (0.79 - 1.28) | OR: 0.87 (0.67 - 1.13) | OR: 0.97 (0.76 - 1.23) | OR: 1.13 (0.86 - 1.47) |  |  |
|  | **Sampling Frame** | | | | | **SES** | | **Utilization by SES Grouping (Number, %)** | | | | | | |
| **Study** | **Country** | **Data Source** | **Study Population^a^** | **Predictive Biomarker Test/ Biological and Precision Therapies Overall Utilization (Number, %)** | **Comparator** | **Unit** | **Measure** | **Lowest SES Group** | | **Highest SES Group** | | | **Utilization in Low SES (OR & 95% CI) ^b^** | **QA** |
| Shih *et al.* (2009)^f^ | USA | NCDB | Captures 75% of Newly Diagnosed Cancer Cases  1998 - 2004  Stage: IV  MCRC  n = 16,027 | Immunotherapy  n = 662 (4.1) | NR | Zip Code | % Without a High School Degree | 29%+ (Low)  OR: 0.60  (0.35 - 1.03)^g^ | 20% - 28.9%  OR: 0.70 (0.45 - 1.07)^g^ | 14% - 19.9%  OR: 0.79 (0.53 - 1.16)^g^ | < 14% (High)  OR: Ref^g^ |  | ^h^ | 7 |
|  |  |  |  |  |  | Zip Code | Median Household Income | < $30,000 (Low)  OR: Ref^g^ | $30,000 - $34,999  OR: 1.09  (0.66 - 1.81)^g^ | $35,000 - $45,999  OR: 0.92  (0.56 - 1.51)^g^ | $46,000+ (High)  OR: 0.92  (0.56 - 1.52)^g^ |  |  |  |
| Taylor *et al.* (2019)* | USA | NCDB | Captures 70% of Newly Diagnosed Malignancies from 1,500+ CoC Facilities  2004 - 2015  Anorectal Melanoma  n = 1,305 | Immunotherapy  n = 221 (16.9) | No Immunotherapy | Zip Code | Median Household Income | < $38,000  (Low)  >25/194(^j^) | $38,000 - $47,999  47/305 (15.4) | $ 48,000 - $62,999  68/359 (18.9) | ≥ $63,000 (High)  74/426 (17.4) |  | 0.87  (0.57 - 1.31) ^k^ | 5.5 |
|  |  |  |  |  |  |  |  | P Value: 0.68 | | | |  |  |  |
|  |  |  |  |  |  | Zip Code | % High School Failure Rate | ≥ 21.0% (Low)  >25/212(^j^) | 13.0% - 20.9%  51/307 (16.6) | 7.0% - 12.9%  79/414 (19.1) | < 7.0% (High)  61/353 (17.3) |  | 0.95  (0.62 - 1.46) ^k^ |  |
|  |  |  |  |  |  |  |  | P Value: 0.41 | | | |  |  |  |
| **Renal Cell Carcinoma: Biological & Precision Therapies** | | | | | | | | | | | | | | |
| Saigal *et al.* (2010)* | USA | SEER - Medicare | 15 US Registries  1992 - 2002  Stage: Metastatic  n = 3,730^c^ | IL-2  n = 560 (15.0) | Radial Nephrectomy/  Both Treatments/  Neither Treatment | Census Tract | Median Income | < $35,000 (Low)  68/970 (7.0)^c^ | $35,000 - $45,000  70/1,007 (7.0)^c^ | $45,000 - $60,000  87/970 (9.0)^c^ | > $60,000 (High)  70/783 (9.0)^c^ |  | 0.77  (0.53 - 1.10) | 4.5 |
|  |  |  |  |  |  |  |  | P Value: 0.3373 | | | |  |  |  |
|  |  |  |  |  |  | Census Tract | % of Non-High School Graduates | > 35% (Low)  22/373  (6.0)^c^ | 20% - 35%  65/933  (7.0)^c^ | 10% - 20%  141/1,567 (9.0)^c^ | < 10% (High)  72/895  (8.0)^c^ |  | 0.72  (0.42 - 1.19) |  |
|  |  |  |  |  |  |  |  | P Value: **0.0394** | | | |  |  |  |
| **Head & Neck Cancer: Biological & Precision Therapies** | | | | | | | | | | | | | | |
| Amini *et al.* (2018)* | USA | SEER - Medicare | 28% of US Cetuximab  Population n = 173 (42.3)  2006 – 2011  Age ≥ 65  Oropharyngeal  Cancer  n = 409 | | Cisplatin/ Carboplatin | Census Tract | Median Income | Lowest (Low)  128/307 (41.7) | Other (High)  45/102 (44.1) |  |  |  | 0.91  (0.56 - 1.46) | 8 |
|  |  |  |  |  |  |  |  | OR (Cetuximab over Cisplatin)  OR: 1.86  (0.93 - 3.73) | OR: Ref |  |  |  |  |  |
|  |  |  |  |  |  |  |  | P Value: 0.079 | |  |  |  |  |  |
|  | **Sampling Frame** | | | | | **SES** | | **Utilization by SES Grouping (Number, %)** | | | | | | |
| **Study** | **Country** | **Data Source** | **Study Population^a^** | **Predictive Biomarker Test/ Biological and Precision Therapies Overall Utilization (Number, %)** | **Comparator** | **Unit** | **Measure** | **Lowest SES Group** | | **Highest SES Group** | | | **Utilization in Low SES (OR & 95% CI) ^b^** | **QA** |
| Xiang *et al.* (2018)* | USA | SEER - Medicare | 17 Registries  (30% of US Population)  2004 - 2013  Age > 65  Stage III - IVB  Oropharynx, Larynx & Hypopharynx Cancers  n = 1,395  n (PSM) = 828 | Cetuximab & Radiotherapy  n = 609 (43.7)  n (PSM) = 414 (50.0) | Cisplatin & Radiotherapy | Census Tract | Poverty Level | > 20% (Low)  128/293 (43.7) | 10% - 20%  154/384 (40.1) | < 10% (High)  327/718 (45.5) |  |  | 0.93  (0.70 - 1.23) | 6 |
|  |  |  |  |  |  |  |  | P Value: 0.22 |  |  |  |  |  |  |
|  |  |  |  |  |  |  |  | PSM:  88/184 (47.8) | PSM:  111/228 (48.7) | PSM:  215/416 (51.7) |  |  | 0.86  (0.60 - 1.23) |  |
|  |  |  |  |  |  |  |  | P Value: 0.61 |  |  |  |  |  |  |
| **Mixed Cancers: Biological & Precision Therapies** | | | | | | | | | | | | | | |
| Hershman *et al.* (2013)* | USA | SEER - Medicare | Registry Coverage: NR  01/2004 - 12/2007  Age > 65  Stage IV or Recurrent  Breast, Colon & NSCLC Cancers  n = 16,085 | Bevacizumab  n = 3,039 (18.9) | No Bevacizumab | Census Tract | SES Score Based on Education, Poverty and Income | 1^st^ (Low)  327/1,870 (17.5) | 2^nd^  514/2,901 (17.7) | 3^rd^  689/3,590 (19.2) | 4^th^  697/3,642 (19.1) | 5^th^ (High)  810/4,065 (19.9) | 0.85  (0.74 - 0.98) | 9 |
|  |  |  |  |  |  |  |  | P Value: 0.08 | | | | |  |  |
|  |  |  |  |  |  |  |  | OR: Ref | OR: 0.97 (0.82 - 1.14) | OR: 1.04 (0.88 - 1.22) | OR: 1.06 (0.90 - 1.25) | OR: 1.09 (0.92 - 1.28) |  |  |
|  |  |  |  |  |  |  |  |  | P Value:  0.75 | P Value:  0.65 | P Value:  0.53 | P Value: 0.33 |  |  |
| Mohile *et al.* (2013)* | USA | Cancer and Aging Research Group’s ‘Determining the Utility of an Assessment Tool for Older Adults with Cancer’ Multi-institutional Trial | 7 Institutions Bevacizumab  2006 – 2009 Plus  Age ≥ 65 Chemotherapy  CRC & NSCLC n = 27 (13.0)  Scheduled to  Receive a New  Chemotherapy  Regimen  n = 207 | | Chemotherapy Alone | NR | Education | Less than 9^th^ Grade (Low)  3/12 (25.0) | 9^th^ Grade/High School  10/80 (12.5) | Some College  9/78 (11.5) | Associate Degree + (High)  5/37 (13.5) |  | 2.13  (0.27 - 13.4) | 6 |
|  |  |  |  |  |  |  |  | P Value: 0.6984 | | | |  |  |  |

^a^ Refers to the total number of patients in the cohorts of interest.

^b^ Author generated.

^c^ Numbers generated from percentage reported data which in some instances may lead to under or over estimations of true patient numbers in each SES measure sub-grouping as well as for the overall study numbers.

^d^ Study reported twice in table due to reporting of both predictive biomarker test and biological and precision therapy data of interest.

^e^ Data extracted for white women only as data for some black women was suppressed where number counts were ≤ 11.

^f^ Study reported twice in table due to separation of results for two cancers of interest.

^g^ OR data is for the ≥ 65 cohort. Data was unavailable to report the ≥ 65 total cohort study population numbers.

^h^ Unable to calculate OR from raw data.

^I^ TKI data prioritized for calculations and meta-analyses (larger population sample with oncogenic driver for therapy in question). This was to avoid potentially counting patients twice, should they have also received bevacizumab.

^j^ Unable to calculate patient numbers receiving targeted treatments due to raw data suppression on account of small numbers. Authors have used a minimum value of 25 to represent >25 for total study population calculation purposes.

^k^ OR calculated from comparison of Q2 to Q4.

* Eligible for inclusion in the meta-analysis.

All individual study OR reported are for predictive biomarker test and biological and precision therapy utilization unless otherwise stated.

Quality appraisal scores range from 0 (lowest) to 10 (highest).

***Italicized & Bolded Studies*** = Paper selected for reporting when multiple publications reporting identical or heavily overlapping study populations presented.

**P Values** = Significant at P < 0.05

Abbreviations: BCLC: Barcelona Clinic Liver Cancer Stage; CALR: Cancer Activity Level Reporting Database; CCR: California Cancer Registry; CI: 95% Confidence Interval; CIHI-DAD: Canadian Institute of Health Information Discharge Abstract Database; CoC: Commission on Cancer; CRC: Colorectal Cancer; CRN: Cancer Research Network; ER; Estrogen Receptor; HER2: Human Epidermal Growth Factor Receptor 2; HMO: Health Maintenance Organization; ICES: Institute for Clinical Evaluative Sciences; IL-2: Interleukin-2; IRR: Incidence Rate Ratios; NACRS: National Ambulatory Care Reporting System; NCCN: National Comprehensive Cancer Network; NCDB: National Cancer Database; NCI: National Cancer Institute; NDFP: New Drugs Funding Program; NR: Not Reported; NSCLC: Non-small Cell Lung Cancer; NYSCR: New York State Cancer Registry; OCISS: Ohio Cancer Incidence Surveillance System; OCR: Ontario Cancer Registry; ODB: Ontario Drugs Benefit; OHIP: Ontario Health Insurance Plan; OR: Odds Ratio; POC: Patterns of Care; PR: Progesterone Receptor; PSM: Propensity Score Matching; QA: Quality Appraisal; RPDB: Registered Persons Database; RR: Risk Ratio; SEER: Surveillance, Epidemiology and End Results Program; SES: Socio-economic Status; TKIs: Tyrosine Kinase Inhibitors.

**Table S3: Study quality appraisal result for the 48 included studies**

| **Cancer** | **Paper** | **Q1** | **Q2** | **Q3** | **Q4** | **Q5** | **Q6** | **Q7** | **Q8** | **Q9** | **Q10** | **Total** |
| --- | --- | --- | --- | --- | --- | --- | --- | --- | --- | --- | --- | --- |
| **Predictive Biomarker Testing** | | | | | | | | | | | | |
| **Testing** | Pensa *et al*. (2009) | 0.5 | 1 | 0.5 | 1 | 1 | 0 | 1 | 0 | 0 | 0.5 | 5.5 |
|  | Lund *et al*. (2010) | 1 | 0.5 | 1 | 1 | 1 | 1 | 0 | 0 | 0 | 1 | 6.5 |
|  | Ferrusi *et al*. (2013) | 1 | 1 | 1 | 1 | 1 | 0.5 | 1 | 1 | 1 | 0 | 8.5 |
|  | de Camargo Cancela *et al*. (2015) | 1 | 0.5 | 1 | 1 | 0 | 0 | 1 | 1 | 1 | 0.5 | 7 |
|  | Greenbaum *et al*. (2017) | 1 | 1 | 1 | 0.5 | 1 | 0.5 | 1 | 1 | 1 | 0.5 | 8.5 |
|  | Rico *et al*. (2016) | 1 | 1 | 1 | 1 | 1 | 1 | 1 | 1 | 1 | 0.5 | 9.5 |
|  | Webster *et al*. (2013) | 1 | 1 | 1 | 1 | 1 | 1 | 1 | 1 | 1 | 1 | 10 |
|  | Enewold *et al*. (2017)^a^ | 1 | 0.5 | 1 | 0 | 0 | 1 | 0 | 0 | 0 | 1 | 4.5 |
|  | Enewold & Thomas (2016)^a^ | 1 | 0.5 | 1 | 0 | 0 | 1 | 0 | 0 | 1 | 0.5 | 5 |
|  | Palazzo *et al*. (2019^a^ | 1 | 1 | 1 | 1 | 1 | 1 | 1 | 1 | 1 | 1 | 10 |
|  | Presley *et al*. (2018) | 1 | 1 | 1 | 0.5 | 1 | 1 | 0 | 0 | 0 | 0.5 | 6 |
| **Biological & Precision Therapies** | | | | | | | | | | | | |
| **Breast** | Du *et al*. (2011) | 1 | 0.5 | 1 | 1 | 0 | 0.5 | 0 | 0 | 0 | 1 | 5 |
|  | Vaz-Luis *et al*. (2015) | 1 | 1 | 1 | 0.5 | 1 | 0 | 0 | 0 | 0 | 0.5 | 5 |
|  | Reeder-Hayes *et al*. (2016) | 1 | 1 | 1 | 1 | 1 | 0.5 | 1 | 1 | 1 | 1 | 9.5 |
|  | Vaz-Luis *et al*. (2016) | 1 | 1 | 1 | 1 | 0 | 1 | 1 | 1 | 0 | 0.5 | 7.5 |
|  | Tsai *et al*. (2017) | 1 | 1 | 0.5 | 0 | 1 | 0 | 1 | 0.5 | 0 | 1 | 6 |
|  | Freedman *et al*. (2013) | 1 | 0.5 | 0.5 | 0 | 1 | 1 | 1 | 1 | 1 | 1 | 8 |
|  | Haas *et al*. (2011) | 1 | 1 | 1 | 0 | 1 | 0.5 | 1 | 1 | 1 | 1 | 8.5 |
|  | Goldhar *et al*. (2016) | 1 | 1 | 0.5 | 0.5 | 1 | 1 | 0 | 0 | 0 | 1 | 6 |
|  | Kumachev *et al*. (2016) | 0.5 | 0.5 | 1 | 0.5 | 0 | 1 | 0 | 0 | 0 | 1 | 4.5 |
|  | Thavendirenathan *et al*. (2016)^b^ & (2018) | 1 | 1 | 1 | 1 | 1 | 0 | 0 | 0 | 0 | 0 | 5 |
|  | Li *et al*. (2018) | 1 | 1 | 0.5 | 0.5 | 1 | 0.5 | 0 | 0 | 1 | 0.5 | 6 |
|  | Shih *et al*. (2009)^a^ | 0.5 | 1 | 1 | 0.5 | 1 | 0 | 1 | 1 | 0 | 1 | 7 |
| **Lung** | Zhu *et al*. (2012) | 1 | 1 | 1 | 1 | 1 | 1 | 0 | 1 | 0 | 1 | 8 |
|  | Langer *et al*. (2014) | 1 | 1 | 1 | 1 | 1 | 0.5 | 0 | 0 | 0 | 0.5 | 6 |
|  | Ritzwoller *et al*. (2014)^b^, Delate *et al*. (2014) & Carroll *et al*. (2015) | 0.5 | 1 | 1 | 1 | 1 | 1 | 0 | 0 | 0 | 1 | 6.5 |
|  | Menter *et al*. (2016) | 0.5 | 0.5 | 0.5 | 1 | 1 | 0 | 0 | 1 | 0 | 1 | 5.5 |
|  | Maguire *et al*. (2019a) & (2019b)^b^ | 1 | 1 | 1 | 1 | 1 | 0 | 1 | 1 | 1 | 0.5 | 8.5 |
|  | Palazzo *et al*. (2019)^a^ | 1 | 0.5 | 1 | 0.5 | 1 | 1 | 1 | 1 | 1 | 1 | 9 |
|  | Enewold & Thomas (2016)^a^ | 0.5 | 0.5 | 1 | 0.5 | 1 | 1 | 0 | 0 | 1 | 0.5 | 6 |
| **Cancer** | **Paper** | **Q1** | **Q2** | **Q3** | **Q4** | **Q5** | **Q6** | **Q7** | **Q8** | **Q9** | **Q10** | **Total** |
| **Lung** | Verma *et al*. (2019) | 1 | 1 | 0.5 | 1 | 1 | 1 | 1 | 0.5 | 0 | 1 | 8 |
|  | Lairson *et al*. (2015) | 1 | 1 | 0.5 | 0.5 | 1 | 0 | 0 | 1 | 0 | 1 | 6 |
| **Hepatobiliary** | Sahara *et al*. (2019) | 1 | 1 | 0.5 | 0.5 | 1 | 1 | 1 | 0.5 | 1 | 0.5 | 8 |
|  | Sanoff *et al*. (2016) | 1 | 1 | 1 | 1 | 1 | 0 | 0 | 0 | 0 | 1 | 6 |
|  | Parsons *et al*. (2016) | 1 | 1 | 1 | 0 | 0 | 1 | 1 | 1 | 0 | 0.5 | 6.5 |
|  | Sarpel *et al*. (2018) | 0.5 | 1 | 1 | 0.5 | 1 | 1 | 1 | 0.5 | 1 | 1 | 8.5 |
| **Melanoma** | Enewold *et al*. (2017)^a^ | 1 | 1 | 1 | 0.5 | 0 | 1 | 0 | 0 | 0 | 0.5 | 5 |
|  | Al-Qurayshi *et al*. (2018) | 0.5 | 1 | 0.5 | 0.5 | 0 | 1 | 1 | 1 | 1 | 0 | 6.5 |
|  | Haque *et al*. (2019) | 1 | 1 | 1 | 1 | 1 | 1 | 1 | 0.5 | 0 | 1 | 8.5 |
| **Colorectal** | Fu *et al*. (2014) | 0.5 | 1 | 1 | 1 | 0 | 1 | 1 | 1 | 1 | 1 | 8.5 |
|  | Cen *et al*. (2012) | 1 | 1 | 1 | 1 | 0 | 0 | 0 | 0 | 0 | 0 | 4 |
|  | Meyerhardt *et al*. (2012) | 1 | 1 | 1 | 0.5 | 1 | 0 | 0 | 0 | 0 | 1 | 5.5 |
|  | Parikh *et al*. (2016) | 1 | 1 | 1 | 0.5 | 1 | 0 | 1 | 0.5 | 0 | 1 | 7 |
|  | Neugut *et al*. (2012) | 1 | 1 | 1 | 0.5 | 0 | 0 | 1 | 1 | 0 | 1 | 6.5 |
| **Colorectal** | Raab *et al*. (2019) | 1 | 0.5 | 1 | 1 | 1 | 1 | 1 | 0.5 | 1 | 1 | 9 |
|  | Shih *et al*. (2009)^a^ | 0.5 | 1 | 1 | 0.5 | 1 | 0 | 1 | 1 | 0 | 1 | 7 |
|  | Taylor *et al*. (2019) | 1 | 0.5 | 1 | 0.5 | 1 | 1 | 0 | 0 | 0 | 0.5 | 5.5 |
| **Renal Cell** | Saigal *et al*. (2010) | 1 | 0 | 1 | 1 | 0 | 1 | 0 | 0 | 0 | 0.5 | 4.5 |
| **Head & Neck** | Amini *et al*. (2018) | 1 | 1 | 1 | 1 | 1 | 0.5 | 1 | 0.5 | 0 | 1 | 8 |
|  | Xiang *et al*. (2018) | 0.5 | 1 | 1 | 1 | 1 | 1 | 0 | 0.5 | 0 | 0 | 6 |
| **Mixed Cancers** | Hershman *et al*. (2013) | 1 | 1 | 1 | 1 | 1 | 1 | 1 | 1 | 0 | 1 | 9 |
|  | Mohile *et al*. (2013) | 0.5 | 1 | 0.5 | 1 | 1 | 1 | 0 | 0 | 0 | 1 | 6 |

Q1: Data sources; Q2: Methods (study population and variables); Q3 & 4: Methods (operational definitions); Q5, 6, 7, & 8: Results and statistics; Q9 & 10: Discussion/conclusions.

^a^Paper reported twice in the table under different cancer sites or included data on predictive biomarkers as well as biologicals and precision therapies.

^b^Paper selected for reporting when multiple publications reporting identical or heavily overlapping study populations presented.

**Figure S2: Sensitivity analyses for biological and precision therapy utilization odds for all cancers (sub-grouped by drug class) for low compared to high socio-economic status.**

Figure S2a: Sensitivity analysis for Figure 3 (main manuscript) substituting included studies (Du *et al.,* (2011), Kumachev *et al.,* (2016), Palazzo *et al.,* (2019), Reeder-Hayes *et al.,* (2016), Sanoff *et al.,* (2016), Fu *et al.,* (2014), Langer *et al.,* (2014) & Menter *et al.,* (2016)) with those excluded due to overlapping sampling frames.

Figure S2b: Sensitivity analysis for Figure 3 (main manuscript) removing the non-USA healthcare setting studies (Kumachev *et al.,* (2016), Li *et al.,* (2018) & Thavendiranathan *et al.,* (2016)).

**Figure S3: Forest plot of odds of biological and precision therapy utilization (sub-grouped by cancer type) for low compared to high socio-economic status (including funnel plot) for all other cancers (apart from breast and lung cancer).**

Hershman et al. (2013) refers to breast, colon and NSCLC cancers.

Mohile et al. (2013) refers to colorectal and NSCLC cancers.

Figure S3: Funnel plot of biological and precision therapy utilization for all other cancer studies.

**Figure S4: Forest plot of odds of biological and precision therapy utilization for all eligible studies (sub-grouped by breast cancer, lung cancer and all other cancers) for low compared to high socio-economic status (including funnel plot).**

Figure S4: Funnel plot of biological and precision therapy utilization for all eligible studies sub-grouped by breast cancer, lung cancer and all other cancers.

**Figure 5: Funnel plots for all other included meta-analyses with ten plus studies.**

Figure S5a: Funnel plot for predictive biomarker test utilization studies (see Figure 2).

Figure S5b: Funnel plot for all cancers’ biological and precision therapy utilization studies (see Figure 3).
